# Formation of artificial chromosomes in *Caenorhabditis elegans* and analyses of their segregation in mitosis, DNA sequence composition and holocentromere organization

**DOI:** 10.1093/nar/gkab690

**Published:** 2021-08-20

**Authors:** Zhongyang Lin, Yichun Xie, Wenyan Nong, Xiaoliang Ren, Runsheng Li, Zhongying Zhao, Jerome Ho Lam Hui, Karen Wing Yee Yuen

**Affiliations:** School of Biological Sciences, the University of Hong Kong, Kadoorie Biological Sciences Building, Pokfulam Road, Hong Kong; School of Life Sciences, Simon F.S. Li Marine Science Laboratory, State Key Laboratory of Agrobiotechnology, The Chinese University of Hong Kong, Hong Kong; School of Life Sciences, Simon F.S. Li Marine Science Laboratory, State Key Laboratory of Agrobiotechnology, The Chinese University of Hong Kong, Hong Kong; Department of Biology, Baptist University of Hong Kong, Sir Run Run Shaw Building, Ho Sin Hang Campus, Kowloon Tong, Hong Kong; Department of Biology, Baptist University of Hong Kong, Sir Run Run Shaw Building, Ho Sin Hang Campus, Kowloon Tong, Hong Kong; Department of Biology, Baptist University of Hong Kong, Sir Run Run Shaw Building, Ho Sin Hang Campus, Kowloon Tong, Hong Kong; School of Life Sciences, Simon F.S. Li Marine Science Laboratory, State Key Laboratory of Agrobiotechnology, The Chinese University of Hong Kong, Hong Kong; School of Biological Sciences, the University of Hong Kong, Kadoorie Biological Sciences Building, Pokfulam Road, Hong Kong

## Abstract

To investigate how exogenous DNA concatemerizes to form episomal artificial chromosomes (ACs), acquire equal segregation ability and maintain stable holocentromeres, we injected DNA sequences with different features, including sequences that are repetitive or complex, and sequences with different AT-contents, into the gonad of *Caenorhabditis elegans* to form ACs in embryos, and monitored AC mitotic segregation. We demonstrated that AT-poor sequences (26% AT-content) delayed the acquisition of segregation competency of newly formed ACs. We also co-injected fragmented *Saccharomyces cerevisiae* genomic DNA, differentially expressed fluorescent markers and ubiquitously expressed selectable marker to construct a less repetitive, more complex AC. We sequenced the whole genome of a strain which propagates this AC through multiple generations, and *de novo* assembled the AC sequences. We discovered CENP-A^HCP-3^ domains/peaks are distributed along the AC, as in endogenous chromosomes, suggesting a holocentric architecture. We found that CENP-A^HCP-3^ binds to the unexpressed marker genes and many fragmented yeast sequences, but is excluded in the yeast extremely high-AT-content centromeric and mitochondrial DNA (> 83% AT-content) on the AC. We identified A-rich motifs in CENP-A^HCP-3^ domains/peaks on the AC and on endogenous chromosomes, which have some similarity with each other and similarity to some non-germline transcription factor binding sites.

## INTRODUCTION

Genetic information is stored in DNA and packaged into chromosomes in all eukaryotic species. To ensure normal functioning of each individual cell and survival of the organism, the integrity of the genome must be maintained. This can be achieved by duplicating all chromosomes accurately and separating them precisely into each daughter cell during every cell division. Errors in chromosome segregation can lead to aneuploidy and recurrent errors can lead to chromosomal instability (CIN).

Centromere function is essential for facilitating faithful chromosome segregation during cell divisions, in which the kinetochore build on the centromere to connect to the mitotic or meiotic spindles. Ectopic centromere formation could cause merotelic attachments, chromosome breakage-fusion-bridge (BFB) cycles and chromosomal rearrangements. Neocentromeres in cancer cells and patients with chromosomal rearrangements are usually found on non-centromeric sequences (1), suggesting that centromeres can be established and maintained epigenetically (2). However, how neocentromere formation was initiated at these ectopic regions is challenging to address, as neocentromeres are often identified way after they are formed (3).

On the other hand, constructing artificial chromosomes (ACs) in many species involves the use of canonical centromeric DNA sequences, like in budding yeast, fission yeast and human cells, suggesting that canonical centromere sequences may have some properties that are preferred for *de novo* centromere formation, at least in these monocentric organisms (4–7). It will be intriguing to further investigate what these favorable properties are, such as AT-richness, repetitiveness, but such preferences may also limit the selection of sequences that can be tested when studying centromere formation. However, *de novo* centromere formation on artificial chromosomes in *C. elegans* embryos do not appear to have stringent sequence requirement (8), thus allowing us to test the efficiency of different DNA sequence features, such as variations in AT-content, repetitiveness and transcription status, in *de novo* formation of centromere and in achieving equal segregation.

When exogenous, naked DNA is injected into the *C. elegans* syncytial gonad, the DNA fragments can be concatemerized into high molecular weight (HMW) DNA arrays in oocytes, followed by chromatinization and *de novo* centromere establishment in embryonic cells (8–11). By co-injection of to-be-tested DNA sequences with a low amount of tandem LacO DNA repeats into a worm strain that expresses GFP::LacI and mCherry::H2B in the germline and embryos, we can monitor the segregation process of newly formed ACs using live-cell time-lapse imaging (8). In this study, we demonstrated that high AT-content sequences, but not repetitive sequences, facilitated the acquisition of segregation capability on newly formed ACs.

The newly formed ACs can also be transmitted to subsequent generations, which allows us to expand the population containing a specific AC to study the average positions of holocentromere domains and potentially any preferred holocentric sequences on a propagated artificial chromosome. Here, we have generated a stably propagated AC in *C. elegans* by co-injection of enzyme-digested budding yeast genomic DNA and several gene markers that are driven by different *C. elegans* promoters with different expression patterns during development. We sequenced the whole genome of this *C. elegans* strain containing this less repetitive and more complex AC, and *de novo* assembled the AC sequences. We mapped CENP-A^HCP-3^ binding sites on this AC, and demonstrated that this stabilized *de novo* holocentromere on the AC is found at unexpressed gene loci on the co-injected marker genes, fragmented yeast sequences. We have identified an A-rich motif, which has with similarity with non-germline transcription factor binding sites, that is enriched in the CENP-A^HCP-3^ domains/peaks on this AC.

Our study has shed light on the concatemerization process of ACs by *in vivo* DNA repair process, the sequence features that facilitates equal AC segregation at the very early embryo stage, and sequence features that are occupied by the centromeric epigenetic mark CENP-A^HCP-3^ in the propagating population. These findings obtained in our *in vivo*, whole organism model will help to elucidate, in a chromosome-wide landscape, how DNA ligation, chromosome formation, chromatinization, *de novo* holocentromere formation and maintenance are regulated.

## MATERIALS AND METHODS

### Random DNA synthesis

1.2-kb long, random DNA sequences with different AT-contents, including 26% AT, 38% AT, 50% AT, 62% AT and 74% AT, were generated by a JavaScript (Random DNA Generator, http://www.faculty.ucr.edu/∼mmaduro/random.htm). Ten random sequences were generated for each AT-content. A 18-bp LacI binding site, LacO (AATTGTGAGCGCTCACAA), was added to both ends (Supplementary Table S1). The sequences were synthesized by Genescript, and the ten random sequences with a specific AT% were combined, and injected as a mix at 100 ng/μl. AC segregation rates were analyzed as described below.

### Plasmid construction

Plasmid WYYp228 consists of a drug selectable marker *Prsp-27::NeoR::unc-54 3′ UTR*, and two fluorescent marker genes, *Pmex5::gfp::h2b::tbb-2 3′ UTR* and *Pmyo-3::mCherry::unc-54 3′ UTR*. *Prsp-27::NeoR::unc-54 3′ UTR*, amplified from the genomic DNA of *C. elegans* strain EG8079; *Pmex5 promoter*, amplified from pJA255 (12); *gfp::h2b::tbb-2 3′ UTR*, amplified from pCFJ420 (13); and *Pmyo-3::mCherry::unc-54 3′ UTR*, amplified from pCFJ104 (14), were inserted into pMD-19T by Gibson assembly (New England Biolabs).

### Time-lapse live-cell imaging and AC segregation assay

To maintain a stable expression level of GFP::LacI, strain OD426 (Supplementary Table S2) was maintained at 22°C. Artificial chromosomes (ACs) were visualized by injecting DNA containing LacO tandem repeats (8,15). Purified plasmid p64xLacO or its linear form (L64xLacO, digested by *Afa*I) was co-injected with to-be-tested DNA molecules (except for the random DNA sequences, which already contain LacO at the ends) into gonads of young adult *C. elegans* as reported (8).

To generate repetitive AC, L64xLacO alone was injected at 100 ng/μl. To increase the sequence complexity of the AC, sheared salmon sperm DNA (91 ng/μl), with a mean size of 5 kb, was co-injected with L64xLacO (9 ng/μl) in a ratio of 10:1.

Injected worms were recovered on MYOB plates seeded with OP50 for 5–8 h before time-lapse live-cell imaging. Threeto four worms were then dissected in 2 μl M9 buffer to release embryos. Embryos were mounted on a freshly prepared 2% agarose pad and the slide edges were sealed with Vaseline. Live-cell images were taken with a Carl Zeiss LSM710 laser scanning confocal microscope with a 16 EC Plan-Neofluar 40× Oil objective lens and PMT detectors. Stacks with 17 × 1.8 μm planes were scanned for each embryo in a 3× zoom and a 1-min or 30-s time interval, with 1.27 μs pixel dwell and 92 μm pinhole. Laser power for 488 nm and 543 nm was set at 5.5% and 6.5%, respectively.

To determine the AC segregation rates, every dividing cell that contains at least one AC was counted as one sample. Each division was categorized as either containing at least a segregating AC, or containing all non-segregating AC(s). Segregating ACs were defined as those that aligned with the metaphase plate and segregated equally with endogenous chromosomes during anaphase. Non-segregating ACs were defined as those that remained in the cytoplasm and did not separate in mitosis. The segregation rate was calculated as the number of dividing cells containing segregating ACs over the total number of dividing cells containing ACs. Among segregating ACs, some may lag and have anaphase bridges, and may not eventually equally divide.

### Construction of a propagated artificial chromosome in *C. elegans*

Salmon sperm DNA (Sigma-Aldrich) was sheared by Covaris M220 to a mean size of 5 kb. Yeast (BY4741) genomic DNA was isolated by the glass beads method (16), followed by double digestion with restriction enzymes, *Afa*I and *Pvu*II. All the fragmented genomic DNA were purified by Qiagen PCR purification kit. The sequences of the three marker genes, NeoR-GFP-mCherry (NGM), are PCR amplified from WYYp228 using primers M13-47 and RV-M (Supplementary Table S3) and purified by Qiagen PCR purification kit. Digested yeast genomic DNA (150 ng/μl) and NGM marker PCR product (5 ng/μl) was co-injected in a ratio of 300:1, and F1 L1 were selected on G418 (25 mg/ml) plates. Three individual adult F1 survivors were singled out repeatedly from each generation and further passed on for 10 generations before analyzing gene marker copy number and whole-genome sequencing.

### Whole worm quantitative polymerase chain reaction (qPCR) and embryo reverse transcriptase qPCR (RT-qPCR)

Five worms from each strain were picked into 50 μl 1× PCR buffer with proteinase K (1 mg/ml; Thermo Fisher) followed by heating at 65°C for 90 min then incubating at 95°C for 15 min to release genomic DNA. 1 μl of worm genomic DNA was used for each qPCR reaction for a final volume of 20 μl.

Embryos from 50 adult worms were collected from bleaching. The embryos were washed three times by M9 buffer before adding 5 μl of worm lysis buffer (5 mM Tris pH 8.0, 0.5% Triton X-100, 0.5% Tween 20, 0.25 mM EDTA and 1 mg/ml proteinase K (Thermo Fisher)), followed by 65°C incubation for 10 min, then 85°C for 1 min.

cDNA synthesis was performed using Maxima H Minus cDNA synthesis kit (Thermo Fisher). 5 μl of cDNA synthesis mix was added to the worm lysate. The final mix contains 1× RT buffer, 0.5 mM each dNTP, 5 μM random hexamer, heat labile dsDNAse 1 unit/μl, RNAse inhibitor and 20 unit/μl reverse transcriptase. The tube was briefly centrifuged, mixed, and incubated at 25°C for 10 min, followed by 55°C for 30 min and finally 85°C for 5 min. The cDNA was diluted to 100 μl with RNase-free H_2_O and 2 μl was used for each PCR reaction for a final volume of 20 μl.

Quantitative PCR was performed using StepOnePlus Real-Time PCR System using the Applied Biosystems™ Fast SYBR™ Green Master Mix with the following parameters: 95°C for 20 s and 40 cycles of 95°C for 3 s, 60°C for 30 s. All data were normalized to the NeoR gene. The primers used are listed in Supplementary Table S3.

### Fluorescence *in situ* hybridization (FISH)

One microgram of *Saccharomyces cerevisiae* (BY4742) genomic DNA were labeled by nick translation to generate green fluorescent probes using Ulysis™ Alexa Fluor™ 488 Nucleic Acid Labeling Kit (ThermoFisher) according to the kit protocol. Adult worms were dissected in 2 μl of 1× egg buffer on a coverslip (25 mM HEPES, pH 7.3, 118 mM NaCl_2_, 48 mM KCl, 2 mM CaCl_2_, 2 mM MgCl_2_) to release embryos. Coverslips on slides were placed in liquid nitrogen followed by freeze-cracking. The embryos were then fixed in –20°C methanol for 30 min. Slides were washed twice in 1× PBS and fixed in PBS with 4% formaldehyde for 10 min. Slides were washed twice in 1× PBS and permeabilized by PBST (0.5% Triton X-100) for 15 min. Slides were washed with 2× SSC for 5 min, followed by incubation with 2× SSC-RNase (2 mg/ml) at 37°C for 45 min. Samples were resuspended in the hybridization mixture (10% dextran sulfate, 2× SSC, 50% formamide, 200 ng/μl sheared salmon sperm DNA, 10 μl probe DNA) at 90°C for 5 min to denature the samples, and followed by incubation in humidified chamber at 37°C overnight. On the next day, samples were washed three times with prewarmed 2× SSC/50% formamide for 5 min at 42°C, followed by three 5-min washes with 2× SSC at 42°C. Wash buffer was removed and followed by immunofluorescence staining, or samples were stained by DAPI (1 μg/ml) at room temperature for 10 min. Mounting was performed using ProLong gold antifade reagent (Life Technologies). The slides were sealed with nail polish and stored at –20°C before imaging.

### Immunofluorescence (IF) staining

Embryos were freeze-cracked after dissection of adult worms and fixed in –20°C methanol for 30 min. After methanol fixation, or after FISH, embryos were then rehydrated in PBS (137 mM NaCl, 2.7 mM KCl, 4.3 mM Na_2_HPO_4_, 1.4 mM KH_2_PO_4_) for 5 min and blocked by AbDil (4% BSA, 0.1% Triton-X 100 in PBS) at room temperature for 20 min. Primary antibody incubation, using rabbit (Rb)-anti-AIR-2 (1:500, a gift from Arshad Desai Lab), Novus Rb-anti-HCP-3 (Q0804, 1:1000 or G3048, 1:2000), was performed at 4°C overnight. Slides were washed with PBST three times for 10 min each. The slides were then incubated with goat-anti-Rb-IgG Cy3-conjugated secondary antibody (1:500; Jackson ImmunoResearch Laboratories, 111-166-045) at room temperature for 1 h, followed by DAPI (1 μg/ml) staining for 15 min. Mounting was done using ProLong gold antifade reagent (Life Technologies).

Images were acquired from Zeiss LSM800 with 40 × 1.4 NA oil DIC, two single PTMs and Airyscan (32-channel GaAsp PMTs). For confocal imaging, embryos or oocytes were captured as z stacks with a z-step size at 0.4 μm, with 30–35 z-sections. Stacks were scanned for each embryo in a 3× zoom, and 3.15 μs of pixel dwell time. DAPI, Alexa 488 and Cy3 channels were scanned with 32 μm pinhole and images were saved in 16 bits format. For Airyscan high resolution imaging, embryos or oocytes were captured as z stacks with a z-step size at 0.18 μm, and 0.62 μs of pixel dwell time. Stacks were scanned for each oocyte in a 6–10× zoom.

### Estimation of the AC size

Grey scale graphs from DAPI staining were used for 3D object segmentation by ImageJ (3D objects counter). Size filter was set to 100–2000 voxels, and threshold pixel value was set to 5500. Signals from yeast FISH probes were used to indicate the AC. Because AC is predicted to be maintained as univalent (2C) in the oocytes while other endogenous chromosomes are bivalent (4C, 400 Mb in total), the size of the AC can be calculated according to the following formula. The integrated density is the sum of the values of the voxels, which are the 3D counterpart of pixels. The ratio of integrated density of AC to other endogenous chromosomes is calculated by the following equation: AC/endo ratio = AC^IntDen^/endo^IntDen^ (6 chromosomes). AC (2C) = AC/endo ratio × 400 Mb. Therefore, AC (1C) = AC/endo ratio × 200 Mb.

### MinION library preparation

The genomic DNA from worm strain WYY35 (Table 1), collected from 3 starved plates, was sequenced using Oxford Nanopore sequencing technology. Briefly, high molecular weight DNA was sheared with a g-TUBE (Covaris) to an average fragment length of around 8 kb. The sheared DNA was repaired using the FFPE Repair Mix, according to the manufacturer's protocol (New England Biolabs). 0.4× Ampure XP beads (Beckman Coulter) was used to exclude short DNA fragments. The DNA ends were blunted and an A overhang was added with the NEBNext End Prep Module (New England Biolabs). Prior to ligation, the DNA solution was cleaned up again by 1× Ampure XP beads. The adapter was ligated to the end-repaired DNA using Blunt/TA Ligase Master Mix (New England Biolabs). The final library was eluted from 0.4x Ampure XP beads after washing 2 times by Adapter Bead Binding buffer (SQK-LSK108 Ligation Sequencing Kit 1D). Two R9.4 Flow-cells were used to sequence the DNA. The MinKNOW software (version 1.5.12) was used to control the sequencing process, and the raw read files were uploaded to the cloud-based Metrichor EPI2ME platform for base calling. Base-called reads were downloaded for further processing and assembly.

**Table 1.** Comparison of the assemblies of the AC by different strategies

	Direct assembly of AC from WGS reads	Assembly of AC after filtering out reads of worm genomic sequence	Assembly of AC after filtering out reads of worm genomic sequence + polish after assembly
**Contig number**	66	50	49
**Total length**	10 922 164 bp	10 813 732 bp	11 072 328 bp
**N50**	418 241 bp	691 347 bp	710 368 bp
**Longest contig**	1 487 696 bp	1 706 458 bp	1 746 823 bp

AC contigs were assembled directly along with endogenous chromosomes, assembled alone after excluding worm genomic sequences, as outlined in Supplementary Figure S4B, or assembled alone plus polishing after assembly by nanopore and Mi-seq reads.

### Mi-Seq library preparation

Genomic DNA from worm strain WYY35 was sheared by Covaris M220 using the default setting for fragment length of 500 bp. About 1 μg of genomic DNA from WYY35 were used for library construction using NEBNext^®^ Ultra™ II DNA Library Prep Kit. The constructed library was run on 1% agarose gel electrophorese for size estimation, and quantified using NEBNext® Library Quant Kit for Illumina®. The library DNA was then diluted to 4 nM and denatured by 0.2 N NaOH for 5 min at 95°C, as described in MiSeq System Denature and Dilute Libraries Guide. Lastly, 12 pM of denatured library DNA was loaded onto the reagent cartridge for the sequencing run. MiSeq sequencing was performed with the assistance from Dr Zhao's lab at Hong Kong Baptist University. Adapter trimming of the raw reads was performed using Trimmomatic (17).

### Embryo fixation and chromatin immunoprecipitation (ChIP)

ChIP experiments were carried out as described in a previous study (18) with slight modifications. In brief, synchronized worm cultures from strain WYY35 were grown at 22°C in batches of 500 ml using 2.8-l flasks and shaking at 230 rpm. The worm liquid culture medium contains the S-Basal Complete, G418 (25 mg/ml) and OP50. Gravid adults were separated from debris by sucrose floating. Two embryo pellet volumes of Embryo buffer (25 mM HEPES–KOH pH 7.6, 118 mM NaCl, 48 mM KCl, 2 mM MgCl_2_, 2 mM CaCl_2_) were added to resuspend the embryos, and chitinase was added at a final concentration of 0.17 U/ml. Chitinase digestion was performed in a 15 ml Falcon tube at room temperature for 30 min and transferred to a 50 ml Falcon tube followed by 2 × 50 ml cool PBS washing. After washing, the embryos were resuspended in 40 ml of cold PBS with 1% formaldehyde. Fixation was performed on ice for 10 min. Excess formaldehyde was quenched by adding 2.2 ml of 2.5 M glycine (120 mM final). Fixed embryos were suspended in 5 volumes of ChIP buffer (50 mM HEPES–KOH pH 7.6, 140 mM NaCl, 1 mM EDTA, 0.5 mM EGTA, 0.5% NP-40 and Protease Inhibitor Cocktail (1 Roche tablet (cOmplete Mini EDTA-free, 11836170001) per 10 ml buffer)). 1 ml suspension was transferred to a milliTUBE (Covaris). Chromatin shearing was performed at 6–10°C with Covaris M220 (settings: Processing Time: 8–20 min; duty cycle: 10%; intensity: 75 W; cycles per burst: 200). The chromatin fragmentation was assessed by the smear pattern between 200 and 500 bp on 1% agarose gel. Debris were pelleted at 10 000 g for 20 min and the supernatant was mixed with 10% glycerol. For each ChIP reaction, 3 mg of protein extract was diluted with ChIP buffer to 900 μl. Then 35 μl 30% sarcosyl (1% final) and 20 μl 5% Na-deoxycholate (0.1% final) were added. 50 μl of the diluted extract were removed (as input sample), mixed with Elution buffer (10 mM Tris–Cl pH 8.0, 1 mM EDTA, 250 mM NaCl, 1% SDS) and processed in parallel with the ChIP samples. 5 μl of antibodies was added (rabbit anti-HCP3, Novus Biologicals Q0804,1 mg/μl) to the chromatin extract and rotated gently at 4°C overnight. On the next day, 50 μl Dynabeads was added to the chromatin extract and rotated for 2 h at 4°C. Beads were washed two times with 1 ml FA Buffer (50 mM HEPES–KOH pH 7.6, 150 mM NaCl, 1 mM EDTA, 1% Triton X-100, 0.1% Na-deoxycholate) for 5 min each, next with 1 ml FA-1000 buffer (50 mM HEPES–KOH pH 7.6, 1 M NaCl, 1 mM EDTA, 1% Triton X-100, 0.1% Na-deoxycholate) for 10 min; with 1 ml FA-500 buffer (50 mM HEPES–KOH pH 7.6, 500 mM NaCl, 1 mM EDTA, 1% Triton X-100, 0.1% Na-deoxycholate) for 10 min, then beads were transferred into a new 1.5-ml Eppendorf tube and washed with another 1 ml FA-500 buffer for 10 min; next with 1 ml TEL buffer (10 mM Tris–HCl pH 8.0, 0.25 M LiCl, 1 mM EDTA, 1% NP-40, 1% Na-deoxycholate) for 10 min, and finally briefly with 1 ml TE buffer (10 mM Tris–HCl pH 8.0, 1 mM EDTA). All washing steps were performed at 4°C. Immunocomplexes were eluted with 50 μl Elution buffer (10 mM Tris–HCl, pH 8.0, 1 mM EDTA, 250 mM NaCl, 1% SDS) for 15 min at 67°C. 120 μl elution buffer was add to the eluted chromatin and input, respectively. All samples were incubated overnight at 65°C to reverse cross-links, then treated with proteinase K (0.44 mg/ml) at 37°C for 2 h. ChIP-ed DNA and the Input DNA were purified by ZYMO ChIP DNA Clean & Concentrator kit. After digestion with RNAse A (0.33 mg/ml) for 2 h at 37°C, DNA from all the samples were purified using ZYMO DNA Clean & Concentrator kit.

### Hi-seq library preparation (for ChIP-seq)

Library preparation and Illumina sequencing (Pair-End sequencing of 101 bp) were performed at the University of Hong Kong, Centre for Genomic Sciences (HKU, CGS). The 4 libraries (two replicates of Input and ChIP-ed DNA) were prepared based on the protocol of KAPA Hyper Prep Kit (KR0961 – v5.16). For each library, 0.8 ng of DNA was performed with reactions of end-repair, 3′ end A-tailing, and indexed adaptor ligation, followed by 16 cycles of PCR amplification reaction for library enrichment. After AMPure beads purification, the quality of each library was analyzed by Agilent Bioanalyzer, Qubit and qPCR. The library was denatured and diluted to optimal concentration and applied in the cluster generation steps. HiSeq PE Cluster Kit v4 with cBot was used for cluster generation on the flow-cell. Illumina HiSeq SBS Kit v4 was used for Pair-End 101-bp sequencing that runs on HiSeq 1500.

### *De novo* genome assembly and evaluation

Adapter trimming of the raw reads was produced from Mi-Seq using Trimmomatic (17). All trimmed pair-end reads were used for *de novo* assembly by SPAdes in its default settings (parameters: -t 24 -m 90 -k 21,33,55,77,99,127 –careful -1).

All MinION reads from two flow cells, despite their quality, were combined and used for Canu1.6 (parameters: genomeSize = 115m useGrid = false overlapper = mhap utgReAlign = true correctedErrorRate = 0.10 -nanopore-raw) nanopore assembly (19). Alternatively, for comparison, high quality reads from the ‘Pass’ category for MinION reads that have a quality score above ‘q6’ were filtered based on a quality metric by Metrichor and used for assembly separately. Highly accurate paired-end reads generated from Mi-seq platform were re-mapped to the assembled contigs. Small indels and misassembles were corrected by Pilon (20). The correcting process was repeated three times until the alignment identity to the reference genome no longer improved. Assemblies from MinION reads were evaluated by dnadiff in MUMmer3 to align the draft assemblies against the *C. elegans* reference genome (WS254). The sequences of AC were assembled by Canu1.6 after filtering out *C. elegans* reads (parameters: genomeSize = 15m useGrid = false overlapper = mhap utgReAlign = true correctedErrorRate = 0.10 -nanopore-raw). The assembled AC contigs were polished by minimap2 + racon for three iterations using nanopore reads, followed by Pilon polishing using Mi-seq reads. The alignment of AC contigs to yeast genome and self-alignment of AC contigs were plotted by D-Genies and YASS (21).

### Chromatin immunoprecipitation followed by DNA sequencing (ChIP-seq) analysis

Paired-end reads from ChIP and input samples were mapped to the reference genome (WS245) using BWA-mem (22). CENP-A^HCP-3^ domains were defined by the MACS2 and automatically called by broad peak (domains) algorithm. Mapped reads from ChIP and input were used to call peaks and obtain read coverage per base using MACS2 under broad domain setting with *P*-value = 1e–3 cutoff. (parameters: -f BAM -g 11072328 -B -p 1e-3 –broad –broad-cutoff 0.1 –fix-bimodal –extsize 100). Log_2_ enrichment ratios (ChIP/Input) of each replicate were calculated using Deeptools software (23). To compare with the published ChIP-chip data (18), the enriched CENP-A^HCP-3^ microarray data were obtained from modENCODE. For comparing the correlation between current ChIP-seq and the previous ChIP-chip, and among ChIP-seq replicates, the average scores of each 1-kb bin over the entire genomic region were calculated. CENP-A^HCP-3^ domains/peaks that are defined by MACS2 were plotted according to their width and AT% by ggplot2 in R. CENP-A^HCP-3^ motif was found by MEME Version 5.3.0 (parameters: -dna -oc. -nostatus -time 18000 -mod anr -nmotifs 3 -minw 6 -maxw 50 -objfun classic -revcomp -markov order 0) (24). Motif-motif similarity was measured by Tomtom (parameters: -no-ssc -oc. -verbosity 1 -min-overlap 5 -mi 1 -dist pearson -evalue -thresh 10.0) (25). Motif sequences from UniPROBE (26) and JASPAR (27) motif database were used to compute the motif similarity.

## RESULTS

### Injection of low AT-content sequence delays the acquisition of AC segregation competency

**Figure 1.**
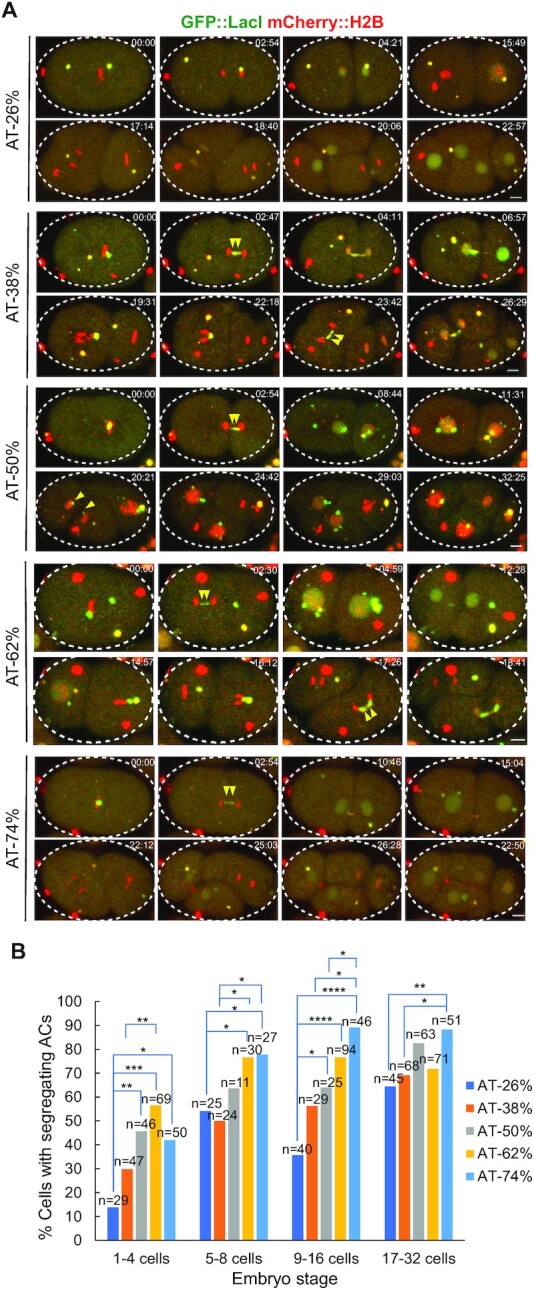
The relationship between AT-contents of injected DNA sequences and AC segregation rates. Ten 1.2-kb long, random sequences were synthesized for each AT-content, including 26% AT, 38% AT, 50% AT, 62% AT and 74% AT. Each sequence has a 18-bp LacI binding site, the LacO sequence (AATTGTGAGCGCTCACAA), at both ends. The 10 random sequences with a specific AT% were combined, and injected as a mix at 100 ng/μl. (**A**) Representative embryos expressing GFP::LacI (green) and mCherry::H2B (red) and carrying ACs with different AT% are shown by live-cell time-lapse imaging. Double yellow arrowheads point to the AC undergoing segregation (either lagging or successfully segregated) from 1-cell to 4-cell stage. The time (mm:ss) is indicated on the top right of images. Scale bar represents 5 μm. (**B**) Quantification of the percentage of cells with segregating ACs, among all dividing cells containing ACs, after injection of synthetic DNA fragment pools with different AT-contents. The number of cells (n) analyzed was indicated. Fisher's exact test was used to test for significance. *P < 0.05, **P < 0.01, ***P < 0.001 and ****P < 0.0001.

To determine the effects of AT-content on new centromere formation, we synthesized ten 1.2-kb random sequences for each of the five different AT%, including 26%, 38% 50%, 62% and 74% of AT. Each synthetic DNA fragment also has a LacI recognition site, the Lac operator sequence (LacO), at both ends. The 10 random sequences with the same AT% were mixed and co-injected into the syncytial gonad of a *C. elegans* strain expressing GFP::LacI and mCherry::H2B. After 6 h of microinjection, embryos were dissected from injected worms for live-cell time-lapse imaging to monitor the segregation rates of the assembled artificial chromosomes (ACs). Representative embryos carrying ACs that were undergoing chromosome segregation from 1-cell stage to 4-cell stage are shown (Figure 1A). We found that in one-cell stage embryos, injected DNA fragments with low AT-content decelerated the ACs to acquire segregation competency in early embryos. The mix of 72% AT segregates better than 26% AT significantly at each embryo stage analyzed (Figure 1B). This trend is consistent when we tested using circular plasmids with three different AT% (Supplementary Figure S1A and B). Our findings suggest that higher AT-content facilitates faster acquisition of segregation capability of ACs in early embryo stage, but such advantage levels off quickly in later embryo stages.

To reveal if repetitive sequences benefit *de novo* centromere formation, we injected linearized p64xLacO sequence (L64xLacO), with or without a complex mix of DNA from sheared salmon sperm DNA (Supplementary Figure S1C). The addition of salmon sperm DNA (10 salmon DNA:1 L64xLacO) allows the formation of ACs with a complex sequence context (28). However, AC segregation analysis shows no significant difference between repetitive and complex ACs, which suggests that repetitive sequences, at least this LacO repeat sequence, cannot facilitate faster acquisition of segregation capability of ACs (Supplementary Figure S1C and D).

### Construction of a propagated artificial chromosome in *C. elegans* using fragmented yeast genomic DNA

To understand how the foreign DNA microinjected to the *C. elegans* gonad is assembled into an artificial chromosome, we constructed an AC with more sequence complexity, and with sequences that can be distinguished from endogenous *C. elegans* sequences. Thereby, genomic DNA from another species was used. We cut the budding yeast *S. cerevisiae* (BY4742, a S288C background strain) genomic and mitochondrial DNA by restriction enzymes, *AfaI* (GT|AC) and *Pvu*II (CAG|CTG), to generate blunt-ended short DNA fragments (50–8448 bp) (Supplementary Figure S2A). The agarose gel electrophoresis image of restriction enzyme-digested budding yeast genomic DNA confirms that the majority of the DNA length is between 100 and 2000 bp (Supplementary Figure S2B).

**Figure 2.**
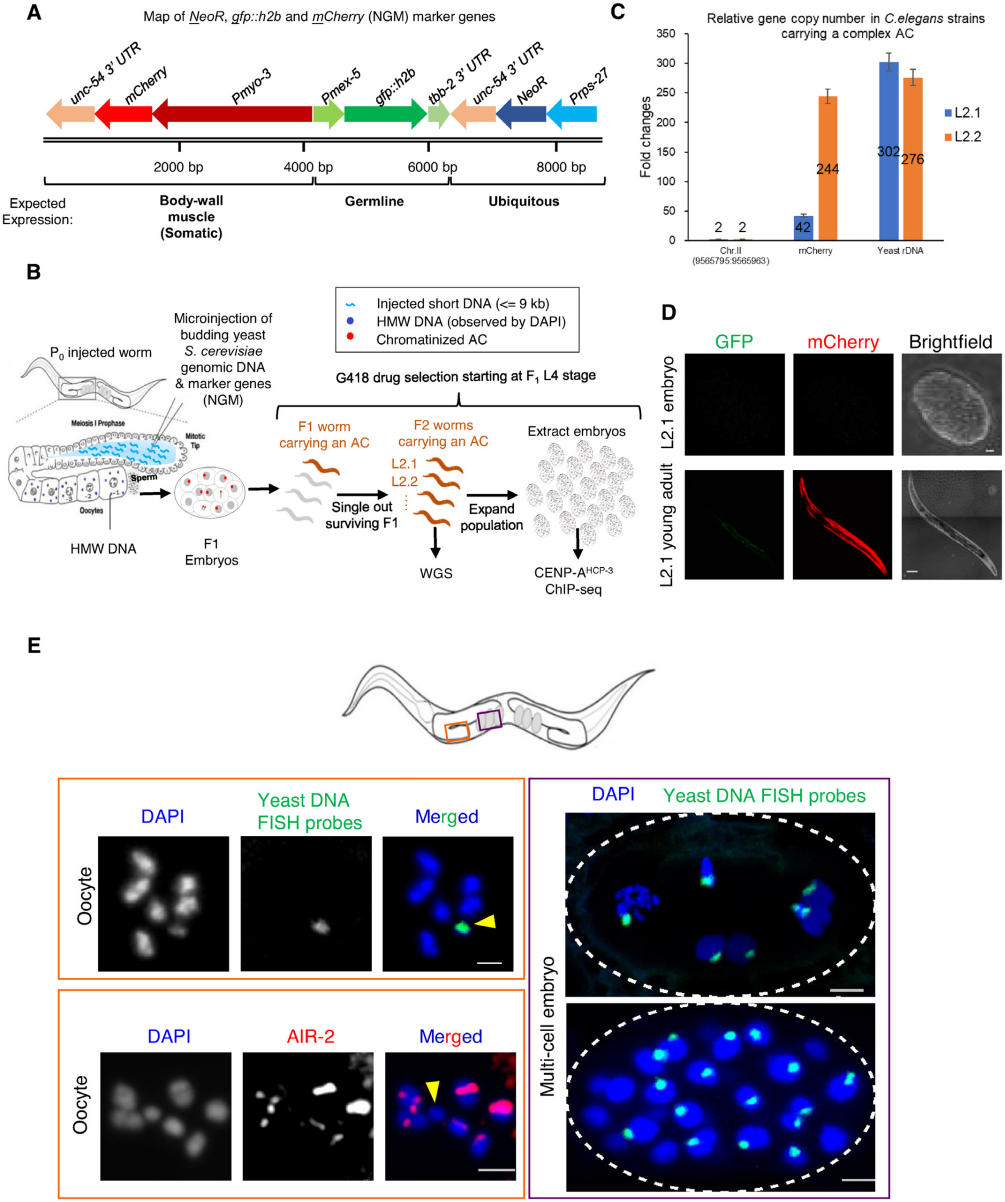
Construction of a complex, propagated artificial chromosome in *C. elegans* using fragmented yeast genomic DNA and marker genes. (**A**) A schematic map of the co-injection markers, *NeoR*, *gfp::h2b*, and *mCherry* (NGM), PCR-amplified from plasmid WYYp228. The purified PCR product (0.5 ng/μl) was used in the co-injection mix with fragmented yeast genomic DNA (150 ng/μl). The antibiotic resistance gene (NeoR) is driven by the ubiquitous *rps-27* promoter. *gfp::h2b* is driven by the germline *mex-5* promoter, and mCherry is driven by the somatic, body wall muscle *myo-3* promoter. The scale bar (in bp) is shown. (**B**) A schematic diagram of the experimental approach used to generate and select a complex, propagated artificial chromosome in *C. elegans* for downstream whole-genome sequencing and chromatin immunoprecipitation followed by sequencing analysis (ChIP-seq). (**C**) Quantification of a unique endogenous locus on Chromosome II (9565795:9565963), mCherry marker, and yeast rDNA copy number in strains (L2.1 and L2.2) with an AC by quantitative PCR. L2.1 and L2.2 are different F2 progenies from the same F1 produced by an injected worm. Genomic DNA was obtained from dauer worms on starved plates. The gene copy number is normalized to the unique endogenous locus, which is assumed to have two copies (before DNA replication) in the diploid organism. Error bars indicate 95% confidence interval (CI) for the mean. (**D**) Representative marker gene expression in embryos and adult worms from strain L2.1. (**E**) The propagated AC (yellow arrowhead) was stained in oocytes and embryos by FISH probes made from yeast genomic DNA. The AC, often smaller than the endogenous chromosomes, lacks AIR-2 signal in the oocytes. DNA was stained by DAPI. In the multi-cell embryos, the propagated ACs aligned at the metaphase plate and segregated with endogenous chromosomes in anaphase and telophase. Scale bar represents 2 and 5 μm in the oocytes and embryos, respectively.

In addition to the yeast DNA, we included three markers genes in the injection. To enable selection for the AC, we express the neomycin resistance gene (NeoR) under the control of the *C. elegans* ubiquitous *rps-27* (a ribosomal subunit) promoter, and *unc-54* (a myosin) *3′ UTR*. To test if different promoter-driven genes are expressed on a complex AC as if on endogenous chromosomes, and if so, how transcription affects holocentromere location, we used a germline expression marker, *Pmex-5::gfp::h2b::tbb-2 3′ UTR*, and a somatic expression marker, *Pmyo-3::mCherry::unc-54 3′ UTR* (Figure 2A). The three genes, NeoR-GFP-mCherry (NGM), were PCR amplified in a linear form from a vector containing these genes. We mixed restriction enzyme-digested yeast DNA (150 ng/μl), with a very low concentration of the NGM marker genes (0.5 ng/μl) (Figure 2A), and co-injected into wild-type (N2) *C. elegans* syncytial gonad.

Injected worms were transferred to new seeded plates to lay eggs. After 2 days, G418 was added onto the surface of each plate of worms for a final concentration of 250 μg/ml. Individual survivors were singled out repeatedly from each generation and further passed on for 10 generations (Figure 2B). To quantify the copy number of NGM markers and specific yeast genomic DNA region, two sublines (L2.1 and L2.2), which were originated from the same F1 progeny with dimmest body wall expression of mCherry, were subject to quantitative PCR (Primers in Supplementary Table S3). We analyzed the copy number of mCherry gene and yeast rDNA. In *S. cerevisiae*, there is a single cluster of rDNA, comprising of approximately 150 copies, located on yeast chromosome XII. This cluster covers about 60% of chromosome XII and about 10% of the whole genome. The fold changes of mCherry and rDNA to a unique worm locus (chr II:9565795–9565963) were used to deduce the copy number of mCherry and rDNA per cell. Subline L2.1 is estimated to contain ∼42 copies of mCherry and ∼302 copies of yeast rDNA per 2C *C. elegans* genome (Figure 2C). The yeast rDNA copy number in L2.1 AC was about two times the copy number of rDNA per haploid yeast genome (Figure 2C) (29). We chose subline L2.1 (WYY35) for further analysis, as L2.1 contains a lower mCherry copy number than subline L2.2, suggesting that L2.1 AC sequences might be more complex than that in L2.2, and could be easier to assemble *de novo*. In L2.1, the germline *mex-5* promoter-driven GFP::H2B signal cannot be observed in germline or embryos, suggesting that it is silenced; whereas *myo-3* promoter-driven mCherry signal can be observed in body wall muscle from L1 to adults, but not in embryos (Figure 2D).

To visualize the propagated AC, we performed DAPI staining, immunofluorescence analysis and fluorescent *in situ* hybridization (FISH) in oocytes and embryos in subline L2.1. DAPI staining together with FISH analysis using DNA probes generated from yeast genomic DNA shows that the AC was not attached to other endogenous chromosomes, but was maintained as an independent chromosomal entity that is well separated from the 6 compact endogenous chromosomes in diakinesis oocytes (Figure 2E and Supplementary Figure S2C). Immunofluorescence staining shows that AIR-2 is absent on the apparently smaller, propagated AC, suggesting that it is a monosomic, univalent chromosome (Figure 2E and Supplementary Figure S2D). To estimate the size of the AC by cytology, the DAPI signal of the AC was compared to that from the 6 bivalent endogenous chromosomes in diakinesis oocytes (Supplementary Figure S2C), which contain replicated diploid genome (4C, ∼400 Mb). Based on the assumption that the propagated AC is fully replicated in oocytes and that the condensation of the AC and endogenous chromosomes are comparable, the size of the AC (1C) was estimated to be about 13 Mb (Supplementary Figure S2C).

Besides, the yeast genomic DNA FISH signals on this AC in multi-cell embryo shows that the AC aligned at the metaphase plate and segregated with endogenous chromosomes in telophase (Figure 2E right panel), suggesting that this AC segregates normally during mitosis. Indeed, CENP-A^HCP-3^ was found on the propagated AC in both oocytes and prometaphase one-cell embryos (Supplementary Figure S2E and F). Because the AC is too close to the endogenous chromosomes in embryos, we were only able to measure the CENP- A^HCP-3^ level in oocytes. The CENP-A^HCP-3^ level on the propagated AC is comparable to that on endogenous chromosomes, at least in oocyte stage (Supplementary Figure S2E).

To estimate cross-generation transmission, we analyzed the percentage of progeny with the presence of body wall mCherry fluorescence. We found that under non-selective condition, the frequency of 3 L2.1 sublines inheriting the AC to the next generation is about 60% (Supplementary Table S4). Based on the FISH images in embryos and the mCherry expression in body wall muscles, the AC is anticipated to be quite mitotically stable. Yet, the univalent, unpaired AC could be lost in female meiosis, similar to the loss rate of the univalent X chromosome in female meiosis in the *him-8 null* background (30).

### DNA sequencing of a *C. elegans* strain with a complex artificial chromosome

To further understand the sequence structure of this propagated AC, we performed whole genome sequencing for strain L2.1 (WYY35). Genomic DNA from strain L2.1 was sequenced directly by nanopore MinION and Mi-seq without PCR to avoid PCR bias during library preparation. Raw data produced from two MinION flow-cells were base-called by Metrichor. Two R9.4 flow-cells ran for 48 h to produce 1 442 172 base-called reads in total, with an average read length of 9804 bases and N50 of 8168 bases (Supplementary Table S5; Supplementary Figure S3A and B). The average yield per flow-cell was about 5.0 Gigabases (Supplementary Table S5). Plotting read length against base-call quality showed no correlation, indicating that there was no quality bias of the read length (Supplementary Figure S3C and D). All reads produced from 2 flow-cells were combined, and the read quality was evaluated by aligning to the *C. elegans* reference genome (WS245). Alignment percentage identity was correlated to the read quality (Pearson, *r* = 0.75) (Supplementary Figure S3E), but not correlated to read length (Pearson *r* = 0.04) (Supplementary Figure S3F). 79.19% of All reads and 88.49% of Pass reads, filtered by Metrichor (Phred quality above 6), were aligned to the *C. elegans* reference genome by Graphmap (31), with a general error rate of 20.73% and 18.87%, respectively (Supplementary Table S6; Supplementary Figure S3G and H). Pass reads showed a higher mappability and lower error rates than All reads.

### *De novo* genome assembly of the *C. elegans* strain with a complex artificial chromosome using MinION and Illumina sequence reads

All reads and Pass reads from MinION were separately assembled by Canu pipelines (19). Assembly from All reads had slightly lower contigs number and slightly higher N50 than assembly from Pass reads (Supplementary Table S7). The alignment of the assembled contigs covered 99.86% of the reference worm genome, without any large gap (Supplementary Figure S4A). Two hundred and forty-one contigs were generated from All reads with N50 of ∼1.49 Mb. Among all the contigs, 86.46% of the contig sequences contain ∼103 Mb with significant homology to 99.85% of the *C. elegans* reference genome, suggesting that the remaining 13.33% of the contig sequences, corresponding to ∼15 Mb, do not belong to the reference worm genome (Supplementary Table S7). Indeed, 66 contigs, containing 10,922,164 bp, were homologous to budding yeast genomic and mitochondrial DNA sequences. Surprisingly, another contig, containing ∼4.77 Mb, belongs to a drug-resistant bacterium, *Stenotrophomonas maltophilia*. The *S. maltophilia* contig is free from yeast or worm sequence. This observation could be due to bacterial contamination on the G418 plates used to culture the nematodes (32). However, we did not identify any reads that belongs to *Escherichiacoli* (OP50), possibly because the worms were isolated from starved plates.

The continuity of *de novo* assembly result of MinION reads by Canu was significantly higher than the assembly of Mi-seq reads using SPAdes, which had a N50 value of only 22 768 bp. However, assembly from the Mi-seq reads had higher 1-to-1 identity to the reference worm genome (99.94%), as compared to 95.84% of the Canu assembly, indicating that Mi-seq reads had higher sequence accuracy (Supplementary Table S7). Thus, Mi-seq reads were only used for polishing the Canu assembly. The percentage identity of Canu contigs to the reference worm genome improved from 95.79% to 99.79% after polishing using the Mi-seq sequence data by applying Pilon (20) three times (Supplementary Table S7).

### *In silico* filtering out *C. elegans* genomic sequences improves the *de novo* assembly result of the AC

As the *C. elegans* genome is already known, direct assembly of the whole genome might be inefficient and not necessary. To establish a straightforward work-flow for AC sequence assembly, we tested if separating AC reads from the *C. elegans* reads first can benefit the *de novo* sequence assembly of AC. To do so, we filtered out reads that belongs to *C. elegans* genome *in silico* and performed *de novo* assembly from the remaining reads (Supplementary Figure S4B). By assembling reads after *in silico* filtering, contig N50 of the AC also improved from 418 241 bp to 691 347 bp, together with a reduction in contig number from 66 to 50 (Table 1). The AC contigs were then further polished by nanopore reads and Mi-seq reads to increase the sequence accuracy. The total length of the polished AC increased to 11 072 328 bp, with N50 of 710,368 bp (Table 1).

### Large DNA fragments from microinjection are preferred to be incorporated into the AC

**Figure 3.**
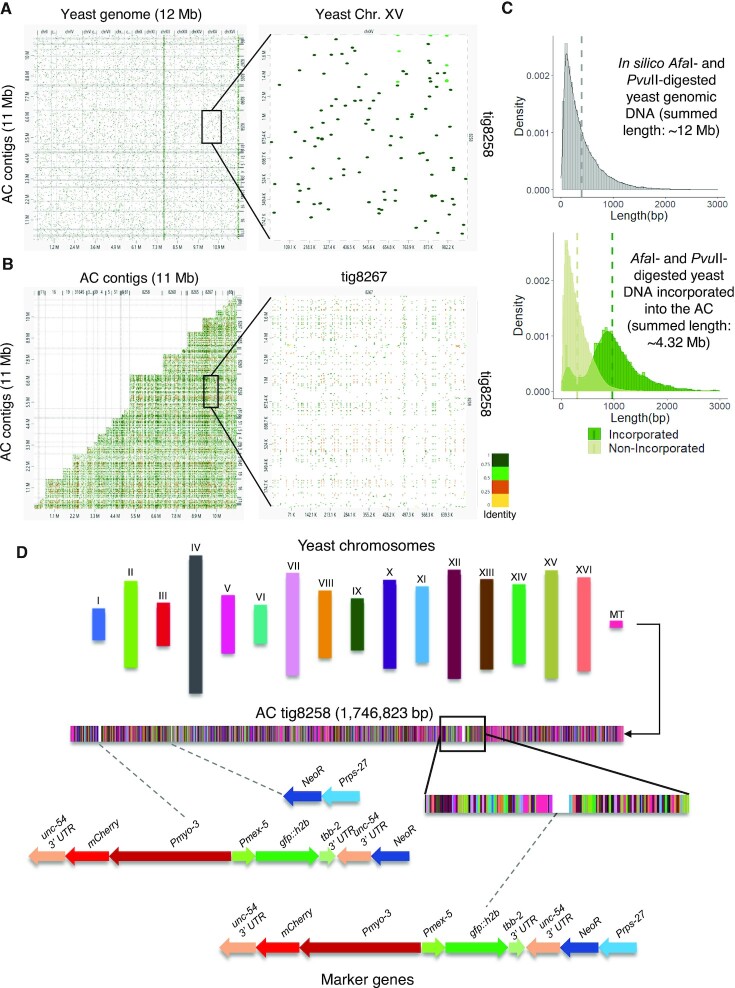
*De novo* assembly results suggested that this AC is mostly formed by random non-homologous end-joining of the injected fragmented yeast genomic DNA and the NGM markers. (**A**) Whole genome alignment of assembled AC contigs to the yeast genome (Chromosome I to XVI) shows that the sequence of each contig is a random combination of short DNA fragments from different yeast chromosomes. The alignment between the largest contig tig8258 and yeast chromosome XV is magnified on the right. (**B**) Self-alignment of the contigs of the assembled AC shows that some short sequences are incorporated multiple times in different or the same contig. The alignment between the largest contig tig8528 and another contig tig8267 is magnified on the right. (**C**) Upper panel: a histogram plot of the DNA fragment lengths distribution density from *in silico Afa*I- and *Pvu*II-digested budding yeast genome (summed-length 12 Mb). Bottom panel: histogram plots of the sequence length distribution density of sequences incorporated into the AC (summed-length ∼4.32 Mb, corresponding to larger fragments) and sequences that did not incorporate into the AC (summed-length ∼7.79 Mb, corresponding to smaller fragments). Dashed lines indicate the mean lengths. (**D**) A schematic representation of the distribution of yeast sequence fragments and the co-injection markers NGM (*Prps-27::NeoR::unc-54 3′ UTR; Pmex5::gfp::tbb-2 3′ UTR; Pmyo-3::mCherry:: unc-54 3′ UTR*) on the largest assembled contig tig8258. DNA fragments belong to individual yeast chromosomes and NGM marker genes are indicated by different colors. The full NGM marker sequences, or fragments inserted are shown in details below.

A comparison between the AC sequences and the yeast genome using dnadiff showed that while this AC size is comparable to that of *S. cerevisiae* genome size (12 Mb), this AC can only align to ∼35% yeast reference sequences, indicating that some sequences were used multiple times in the assembled AC. Genome scale alignment of the assembled AC contigs to the yeast reference genome (S288C) showed that the sequence of each contig was a combination of DNA fragments from each yeast chromosome (Figure 3A). AC self-alignment reveals that some enzyme-digested yeast DNA sequences were used multiple times on the AC (Figure 3B), indicating that the incorporation process of the injected yeast fragmented DNA is not even.

Based on the comparison of *in silico* digested yeast sequences and the AC sequences, we separated enzyme-digested yeast fragments into incorporated and non-incorporated sequences, and noticed their sequence length distribution, respectively. The mean sequence length of the restriction enzyme-digested yeast DNA is about 402 bp (Figure 3C upper panel). However, the incorporated sequences have a mean length of 973 bp, which is ∼3-fold higher than the mean length of non-incorporated sequences (311 bp) (Figure 3C lower panel). This finding indicates that sequences incorporated into the AC are length-biased. Indeed, DNA with length below 500 bp was rarely incorporated into the AC, despite the fact that these short fragments were highly abundant in the injection mixture (Supplementary Figure S2A). In contrast, larger DNA molecules have higher chances to be incorporated in the AC.

To further confirm this phenomenon, we microinjected a 300-bp linear DNA fragment with eight copies of LacO (L8xLacO), at the same concentration (100 ng/μl) as we had used for microinjection of L64xLacO, to the *C. elegans* gonad. By live-cell time-lapse imaging, we found that microinjecting L8xLacO only generated tiny ACs (based on GFP::LacI foci sizes), which cannot equally segregate even in late embryo stage (Supplementary Figure S5A). This result, using just one single sequence, is consistent with the above using different yeast DNA fragment sizes, both suggesting that short DNA sequences were inefficient in forming ACs.

From the alignment of enzyme-digested yeast DNA to the AC sequences, we found that the digested yeast DNA fragments from each yeast chromosome were randomly fused together (Figure 3D and Supplementary Figure S5B). Next, we tried to identify the position of NGM makers. The NGM co-injection markers were found to be interspaced as single copies in between yeast genomic DNA, but not concatemerized in tandem. For example, on the largest contig (tig8258), NGM markers and some of the incomplete NGM fragments were interspaced in between the yeast genomic DNA (Figure 3D and Supplementary Figure S5C). On the NGM sequences, the two *unc-54 3′ UTR* sequences (∼760 bp) are ∼1.6 kb apart. The homologous sequences are sufficiently long and close enough for concatemerizing through HR. However, we have not found any two NGM markers that are tandemly oriented on the assembled contigs (Supplementary Table S8, Figure 3D and Supplementary Figure S5C).

To confirm if non-homologous end-joining (NHEJ) is important for AC formation from linear DNA, we co-injected restriction digested yeast genomic DNA (100 ng/μl) with L64xLacO (10 ng/μl) to the *C. elegans* gonad in wild-type and *lig-4* RNAi-treated embryos (Supplementary Figure S5D). We found that *lig-4* depletion significantly reduces the size of ACs formed in one-cell embryos (Supplementary Figure S5E).

### The holocentromere on the propagated, complex AC is maintained as dispersed CENP-A^HCP-3^ nucleosomes

Both our previous and current study have shown that the propagated AC from injected foreign DNA can establish functional centromeres (8,11) (Supplementary Figure S2E and F). To investigate where the holocentromere localizes on the propagated AC, we performed ChIP-seq to investigate the centromere position on this propagated, complex AC.

**Figure 4.**
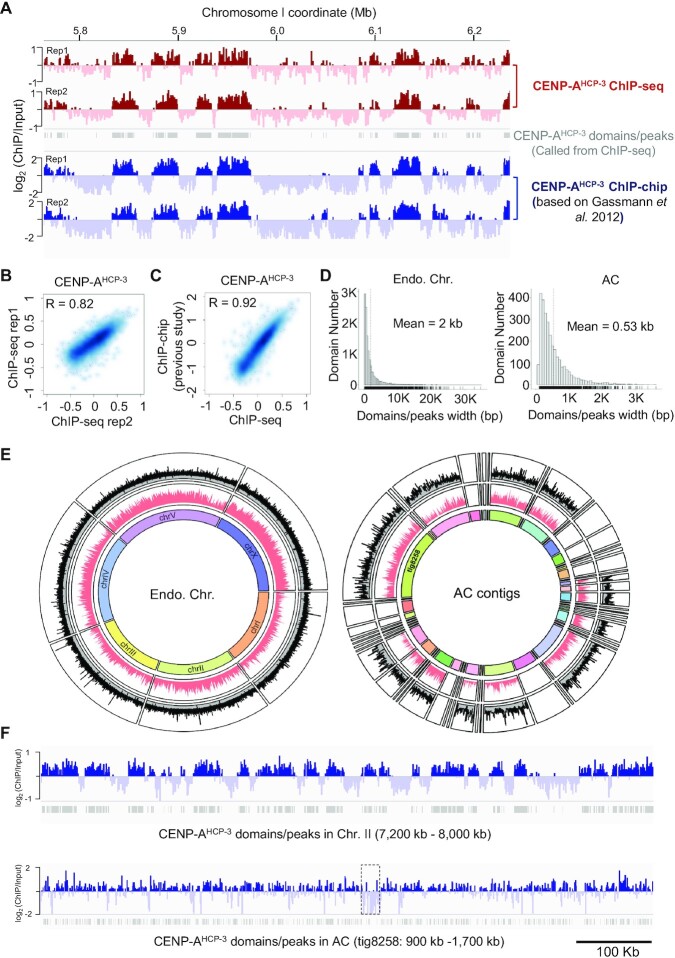

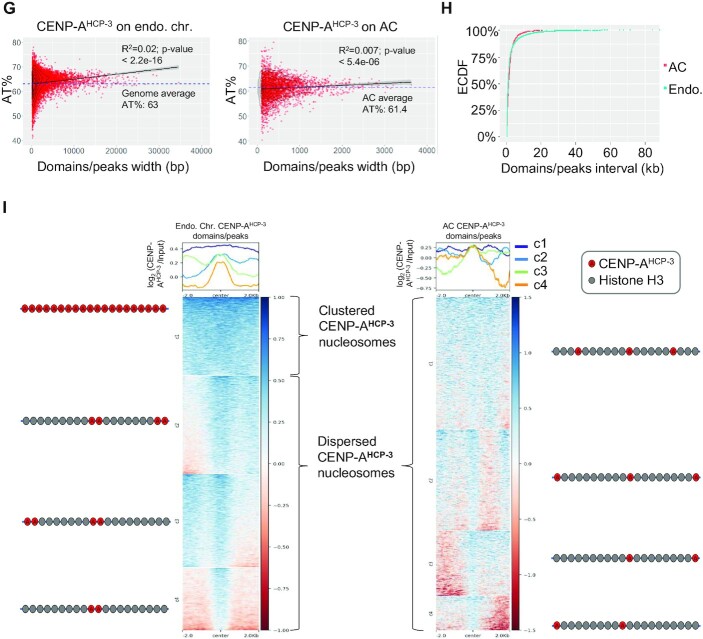
The CENP-A^HCP-3^ domains/peaks on the endogenous chromosomes and on the prorogated AC. (**A**) Representative enrichment of CENP-A^HCP-3^ and CENP-A^HCP-3^ domains/peaks on chromosome I by current ChIP-seq replicates and previous ChIP-chip replicates. (**B**) The localization of CENP-A^HCP-3^ enrichment on endogenous chromosomes between two ChIP-seq replicates are highly correlated. (**C**) The localization of CENP-A^HCP-3^ enrichment on endogenous chromosomes detected by ChIP-seq is highly correlated to previous ChIP-chip results (18). The signal for each data set represents the average of two independent replicates. (**D**) Histogram plots of CENP-A^HCP-3^ domain/peak sizes in endogenous chromosomes (left panel) and in AC (right panel). (**E**) Circos plot of CENP-A^HCP-3^ domains/peaks on all endogenous chromosomes (left panel) and on all 49 AC contigs, including the longest contig tig8258 (right panel). The outside circle shows the CENP-A^HCP-3^ domains/peaks called by MACS2. Y-axis is the signal value (overall enrichment) for the domains/peaks. The inside circle indicate the CENP-A^HCP-3^ domains/peaks density in 10-kb windows. (**F**) Representative CENP-A^HCP-3^ enrichment in a 800-kb region in endogenous Chromosome II (7200–8000 kb) and in AC contig tig8258 (200–1000 kb). CENP-A^HCP-3^ domains/peaks were indicated as grey bars. The dashed box (1 319 126–1 347 939 bp) indicates the region enriched with yeast mitochondrial DNA (the zoom-in is shown in Figure 5A). (**G**) Dot plots of AT-content (%) of each CENP-A^HCP-3^ domain/peak against its size in endogenous chromosomes (left panel) and in AC (right panel). The 2D density plots were indicated by contour lines (bin = 7). The linear regression line to the scatter plot model was shown with 95% confidence region (grey shading). The average *C. elegans* genomic and AC AT-contents are indicated by the blue dotted lines, respectively. (**H**) The empirical cumulative distribution functions (ECDF) of interval distances between CENP-A^HCP-3^ domains/peaks on endogenous chromosomes (blue) and AC (red). (**I**) The CENP-A^HCP-3^ enrichment profiles and heatmaps of the 2-kb flanking regions surrounding the center of each CENP-A^HCP-3^ domain/peak for endogenous chromosomes and AC. Consensus CENP-A^HCP-3^ domains/peaks are separated into four clusters by k-means clustering. The profile plots (upper panel) show the average ChIP signals of each cluster. The ChIP signals of each domain/peak are shown in the clustered heatmaps (lower panel), in which the color coding on the right indicates the log_2_ CENP-A^HCP-3^ ratio. The schematic on the left and right show the possible distribution of CENP-A^HCP-3^ nucleosomes interspaced in between H3 nucleosomes.

As cross-linked ChIP-microarray (ChIP-chip) against CENP-A^HCP-3^ on *C. elegans* endogenous chromosomes has been reported previously (18), we showed a representative genome browser view on 0.5 Mb on chromosome I from current CENP-A^HCP-3^ ChIP-seq and previous ChIP-chip (18), indicating similar patterns of CENP-A^HCP-3^ signals on the chromatin (Figure 4A). We evaluated the reproducibility between our biological replicates of CENP-A^HCP-3^ ChIP-seq, and between current ChIP-seq and previous reported CENP-A^HCP-3^ ChIP-chip results (1 kb bins). We demonstrated that the log_2_ ratio of CENP-A^HCP-3^ between two current ChIP-seq replicates were highly correlated (*r* = 0.82), indicating that our ChIP-seq experiments against CENP-A^HCP-3^ were reproducible (Figure 4B). The cross-platform correlation between pairs of replicates of ChIP-chip and ChIP-seq were also high, with median *r* = 0.92, indicating that CENP-A^HCP-3^ ChIP-seq results corroborate previous CENP-A^HCP-3^ ChIP-chip results (Figure 4C). The mean width of CENP-A^HCP-3^ domains/peaks on endogenous chromosomes is 2 kb, but that on the AC is only 530 bp (Figure 4D). All CENP-A^HCP-3^ domains/peaks identified by MACS2 were plotted, as well as the domains/peaks density (Figure 4E). Small contigs, consisting of 19.71% AC sequences, were excluded from downstream analysis due to low ChIP-seq mapping coverage and failure to call any domains/peaks. We found that CENP-A^HCP-3^ domains/peaks on endogenous chromosomes occupy about 25.39% of the *C. elegans* genome. For the propagated AC, CENP-A^HCP-3^ domains/peaks occupy about 17.62% of the AC sequences (excluding contig sequences that fail to call any domains/peaks), which is slightly lower than the percentage in endogenous chromosomes.

Representative snapshots of the CENP-A^HCP-3^ enrichment on an 800-kb region, on chromosome II (7200–8000 kb) and contig tig8258 (200–1000 kb) of the AC, are also shown (Figure 4F). We found that there is a weak, but significant correlation of CENP-A^HCP-3^ domain/peak width with their AT-contents on endogenous chromosomes, but the correlation is even weaker on the AC (Figure 4G). The majority (∼85%) of interval distance between CENP-A^HCP-3^ domains/peaks on endogenous and artificial chromosomes are similar, except that on endogenous chromosomes, 1.65% of interval distance is more than 20 kb (Figure 4H).

We generated CENP-A^HCP-3^ domain/peak heatmap profiles on the endogenous chromosomes and on the ACs by aligning the center of each domain/peak and plotting the CENP-A^HCP-3^ signals within the 4-kb-flanking region. We observed that the average CENP-A^HCP-3^ profile of endogenous chromosomes displayed two subpeaks, which spans ∼500 bp (Supplementary Figure S6, left panel), suggesting that there could be two CENP-A^HCP-3^ nucleosomes at these domains/peaks. In comparison, the average CENP-A^HCP-3^ profile on the AC has a single peak and is narrower, spanning about 250 bp (Supplementary Figure S6, right panel), suggesting that there might be a single CENP-A^HCP-3^ nucleosome at the domains/peaks on the AC.

We separated CENP-A^HCP-3^ regions into four clusters by the *k*-means algorithm. From the endogenous chromosomes, the CENP-A^HCP-3^ regions can be grouped into clustered domains (covered more than 4 kb, cluster 1) and dispersed CENP-A^HCP-3^ peaks (distinguishable < 500-bp regions, cluster 2–4) (Figure 4I, left panel). In comparison, the majority of CENP-A^HCP-3^ nucleosomes on the AC are in dispersed peaks, which might represent that CENP-A^HCP-3^ nucleosomes on the AC are interspersed between H3 nucleosomes (Figure 4I, right panel).

### Identification of an A-rich CENP-A^HCP-3^ motif on the propagated AC

**Figure 5.**
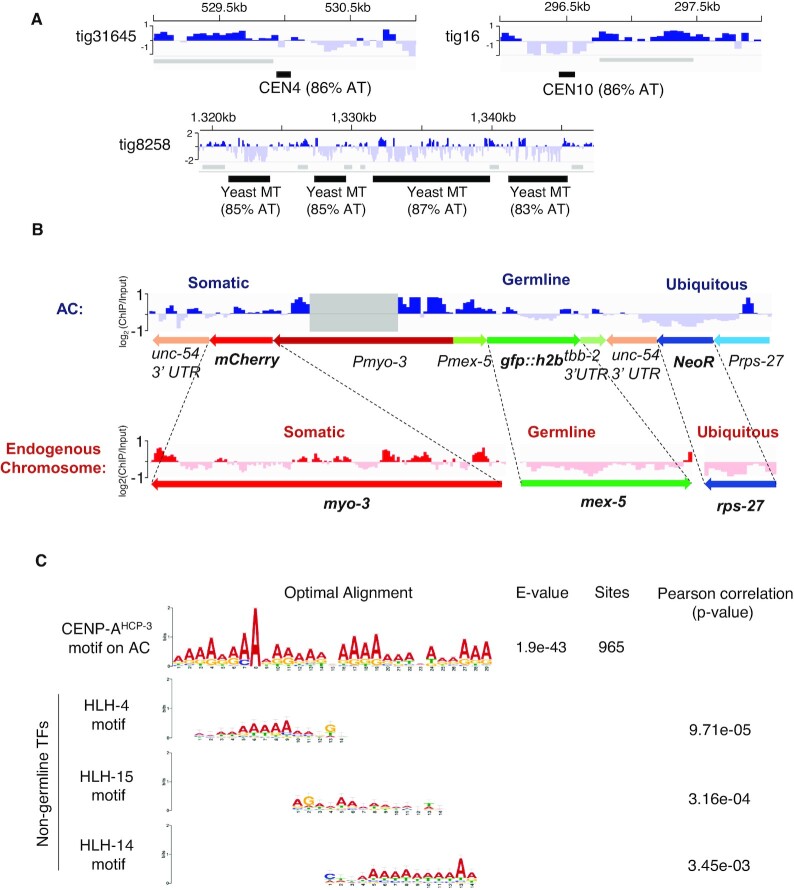
The CENP-A^HCP-3^ distribution pattern on the propagated AC. (**A**) CENP-A^HCP-3^ does not localize on the yeast centromeric sequences, CEN4 and CEN10, and yeast mitochondrial DNA (MT) (the dashed box in Figure 4F) in the propagated AC. CENP-A^HCP-3^ domains were indicated as grey bars. (**B**) The log_2_ CENP-A^HCP-3^ ratio on marker genes is averaged from 10–29 copies (Supplementary Table S9). CENP-A^HCP-3^ on the AC is enriched in the somatic mCherry marker, partially excluded in the silenced germline *gfp::h2b* region, and excluded in the ubiquitous *NeoR* drug resistance gene marker. The corresponding endogenous genes with the same promoters were analyzed and shown in parallel. Non-distinguishable reads that mapped to both endogenous and AC promoter or 3′ UTR regions were excluded from the analysis and the region with all reads removed was shaded. (**C**) The sequence of a 29-bp CENP-A^HCP-3^ motif: AAAARRAARARAADVAAAAAAARARRAAA, is identified at 965 sites on the AC, where R represents A or G; D represents A or G or T; V represents A or C or G. The e-value estimates the expected occurrence of motifs with the same size and frequency in a similarly sized set of random sequences. Toggle error bars indicate the confidence level of a motif based on the number of sites used in its creation. The motifs found in the UniPROBE database with a significant match to the CENP-A^HCP-3^ motif on the AC are shown. The *P*-value indicates the significance of the similarity between CENP-A^HCP-3^ motif and TF motif.

Furthermore, we evaluated the CENP-A^HCP-3^ pattern on the propagated AC. Unlike in budding yeast, in which CENP-A^CSE-4^ is only deposited on the AT-rich, 125-bp centromeric DNA, specifically on CDEII (33), CENP-A^HCP-3^ could deposit on the worm AC comprising of fragmented yeast sequences. Interestingly, we found that CENP-A^HCP-3^ signal is negative on the two yeast centromeric sequences, CEN4 and CEN10, both 84% AT, found on the AC (Figure 5A). This suggests that the yeast centromere sequences are not preferred in *C. elegans* holocentromere. Similarly, yeast mitochondrial DNA sequences, with 83–87% AT, are also devoid of CENP-A^HCP-3^ (Figure 5A).

In addition to checking the marker gene expression by fluorescent images (Figure 2D), we have confirmed the transcription level of each marker gene from early-stage embryos in strain L2.1 by reverse transcription qPCR. We normalized the transcription level of each marker gene to their copy number in AC and compare to NeoR. We found that the transcription level of GFP and mCherry is 0.21-fold and 0.54-fold of that of NeoR, respectively (Supplementary Figure S7A). In endogenous chromosomes, CENP-A^HCP-3^ domains were generally excluded from ubiquitous, embryonic and germline expression regions (18). Consistently, in the propagated AC, CENP-A^HCP-3^ is depleted in the ubiquitous NeoR gene marker, and is enriched in the somatic mCherry marker, which is expressed in body wall muscles and not in embryos (Figure 5B). Because *C. elegans* germline tends to silence exogenous DNA (34), the germline GFP::H2B marker on this complex AC was also silenced in this strain (Figure 2D). Interestingly, CENP-A^HCP-3^ on the germline GFP::H2B marker is half enriched (in which this half is upstream of the somatic mCherry marker) and half depleted (in which this half is downstream of the ubiquitous NeoR gene) (Figure 2A and 5B). Therefore, the CENP-A^HCP-3^-positive and CENP-A^HCP-3^-negative signals on the coding regions of marker genes (averaged from 10 to 29 copies, Supplementary Table S9) were consistent with the pattern on the endogenous genes driven by the same promoters (Figure 5B). Our finding further suggests that CENP-A^HCP-3^ could be negatively correlated to the transcription level of the gene locus in the embryos, and could be influenced by neighboring site enrichment. We did not directly compare the sequence of promoter or 3′ UTR on the AC, because these sequences are identical in endogenous chromosomes and are not distinguishable by the mapping software (Figure 5B).

Based on the sequences of CENP-A^HCP-3^ domains/peaks on this AC and using MEME analysis, we have identified a CENP-A^HCP-3^ motif with high adenine enrichment, AAAARRAARARAADVAAAAAAARARRAAA (Figure 5C). This adenine-rich motif has a significant match to several transcription factor (TF) binding sites in the UniPROBE database, like HLH-4, HLH-15 and HLH-14 binding sites, with *P*-values of 9.71e–05, 3.16e–04 and 3.45e–03, respectively (Figure 5C). Interestingly, HLH-4, HLH-15 and HLH-14 are expressed in neuronal cells and not in the germline or embryos (35,36). Other motifs for non-germline expressed TFs, like BLMP-1 and EOR-1 (from JASPAR database), also have significant matches to the CENP-A^HCP-3^ motif in the propagated AC (Supplementary Figure S7C). Yet, this A-rich motif also matched with more than 85,000 sites in the CENP-A^HCP-3^-negative regions, so it may not be sufficient to determine CENP-A^HCP-3^ occupancy. Analogously, based on the sequences of CENP-A^HCP-3^ domains/peaks on endogenous chromosomes, which occupy less than 500 bp, we found an A-rich motif, RAAAAWKNDGAAAAA, with significant sequence similarity to the A-rich motif on AC centromere (*P*-value: 3.39e–04) (Supplementary Figure S7C). The A-rich motif from endogenous holocentromere also significantly matched with non-germline transcription factor binding sites, including motifs of SKN-1, BLMP-1 and EOR-1 (Supplementary Figure S7D). In comparison, two motifs, SSSVCMMGSGARCCGGAACCCCGAAAGGA and YYATTKCCBCAMCYTTYCCTCTGYHYRMWTYCRAYCGTCCT, identified from the CENP-A^HCP-3^-negative regions on the AC has relatively higher GC%, and has sequence similarity to HLH-2 motif (Supplementary Figure S7E). HLH-2 has been found to express in the proximal gonad (37) and early embryos (38).

## DISCUSSION

AT-rich and repetitive sequences are the common features in most regional monocentromeres and some holocentromeres (39,40). In *C. elegans*, holocentromeres can be rapidly established on microinjected foreign DNA in the absence of any worm genomic DNA sequence (8,41). We used the GFP::LacI-LacO DNA tethering system to visualize newly formed artificial chromosomes containing LacO repeats. By microinjection of DNA with different AT-contents, or in repetitive or complex contexts, we tested the preference of AT-content and sequence repetitiveness in holocentromere establishment on *C. elegans* artificial chromosomes.

High AT-content sequences were routinely found in point monocentromeres, like in *S. cerevisiae*, and in many regional monocentromeres, such as in *S. pombe*, *Drosophila* and humans (39). The *S. cerevisiae* point centromeres’ function are strongly dependent on the high AT-content CDEII sequence (5). H3 nucleosome occupancy is known to be affected by the combination of DNA sequences and chromatin remodeling factors (42). AT-rich or poly (dA:dT) sequence may have a lower affinity for the H3 nucleosome (43), but is preferred for CENP-A nucleosome deposition (44). Ectopic human neocentromeres show some similar features as in native centromeres, in which the CENP-A-binding domains on neocentromeres had slightly higher AT-contents as compared to that of the total genomic DNA (45).H3 nucleosome occupancy is known to be affected by the combination of DNA sequences and chromatin remodeling factors (42). AT-rich or poly (dA:dT) sequence may have a lower affinity for the H3 nucleosome (43), but is preferred for CENP-A nucleosome deposition (44). Ectopic human neocentromeres show some similar features as in native centromeres, in which the CENP-A-binding domains on neocentromeres had slightly higher AT-contents as compared to that of the total genomic DNA (45). We hypothesized that low AT-content sequences may affect the chromatin conformation and disturb CENP-A nucleosome deposition, therefore decelerated AC segregation rate in early embryonic stage. In this study, we tested this hypothesis by comparing the mitotic segregation rates of newly formed ACs generated from input DNA with different AT-contents. We found that ACs with medium and high AT-content 50%, 62% and 74% acquired segregation competency significantly faster than low AT ACs (26%) in 1–4 cell stage embryos, suggesting that the lower AT-content sequences may be more inefficient in recruiting centromeric proteins than medium and high AT-content sequences (Figure 1A and B). However, the disadvantage of medium AT-content (e.g. ∼50, ∼60%) diminishes in a few cell cycles, and the segregation rates catch up with that of higher AT-contents (74%) by 32-cell stage, suggesting the improvement over each cell cycle could be more significant in the long run than the effect of input DNA sequences (Figure 1B and Supplementary Figure S1B). What indeed makes the AC segregation improves over time is intriguing yet puzzling. Our observation suggests that monocentric and holocentric centromeres may share some common DNA features and determining factors for centromere establishment. Currently, it is not clear if the advantage of AT-rich sequences in holocentromere establishment is related to the A-rich motif preference in stable CENP-A^HCP-3^ domains in holocentromere maintenance (Figure 5C and Supplementary Figure S7C).

As for stably propagating holocentromeres, genome-wide analysis of CENP-A^HCP-3^ domains from ChIP-seq shows a correlation of CENP-A^HCP-3^ domain width with AT-rich sequences on endogenous holocentromeres, though this correlation is minimal on newly established holocentromeres (on ACs). This observation suggests that CENP-A^HCP-3^ may tend to accumulate, or be more stable at higher AT-content regions, such that larger CENP-A^HCP-3^ domains could be formed and maintained. However, the extremely high AT% yeast CEN4, CEN10 (86% AT) and mitochondrial DNA regions (83–87%) surprisingly have no CENP-A^HCP-3^ enrichment, suggesting that there may also be an upper AT% limit for *C. elegans* CENP-A^HCP-3^ domains (Figure 5A). Indeed, none of the CENP-A^HCP-3^ domains identified in either AC or endogenous chromosomes exceeds 80% AT (Figure 4G).

Tandem repeats are another common sequence context of human centromeres (alpha satellite repeats), mice centromeres (minor satellite repeats) and fission yeast centromeres (central core repeats), and they have a high competency for *de novo* centromere formation in these organisms (6,7,46,47). In the holocentric plant, *Rhynchospora*, CENP-A also localizes on centromeric repeats (40). However, in holocentric *C. elegans*, centromeric-specific repeats have not been found in endogenous chromosomes (18). To determine the effects of sequence repetitiveness on new centromere formation, AC segregation frequencies were compared between repetitive ACs (made of just linear 64xLacO repeats) and complex ACs (made of a low concentration of linear 64xLacO plus a higher concentration of salmon sperm DNA). Yet, such particular repetitive and complex ACs showed similar segregation rates in each embryonic stage, indicating that sequence complexity may not influence *de novo* holocentromere formation in *C. elegans* (Supplementary Figure S1C and D). More repetitive sequence combination could be tested in the future. Yet, our result showing no preference to repetitive sequence in newly formed AC segregation is consistent with the endogenous holocentromere localization in *C. elegans*, which covers 50% of the genome, mainly on non-repetitive sequences (18).

To compare the *de novo* centromere on the AC and that on the endogenous centromeres, we generated a propagated AC in *C. elegans* made mostly out of yeast genomic DNA. We demonstrated that this AC was maintained as an independent chromosome (Figure 2E), has established centromere (Supplementary Figure S2F) and can align at metaphase plate and segregate with the endogenous chromosomes in mitosis (Figure 2E). However, we found that without drug selection, the average frequency of AC being inherited to the next generation is about 60%. The inheritance of this AC, as other extrachromosomal ACs, between generations does not obey Mendel's law (9,10). We speculated that ACs may not be stably inherited in meiosis. Indeed, the Aurora B kinase, AIR-2, that localizes to the middle region of bivalent chromosomes in the diakinesis oocytes (48), is absent on the AC (Figure 2E and Supplementary Figure S2D), suggesting that the propagated AC is a univalent chromosome in oocytes. Unpaired univalent chromosome tends to be missegregated, and be excluded to the polar body during female meiosis (30), which may explain why ACs are relatively mitotically stable, but are often lost between generations (8–10).

To investigate the DNA concatemerization events that generate this AC, we have applied the Nanopore MinION and Illumina Mi-seq platforms to sequence the whole genome (WGS) of this complex AC-containing *C. elegans* strain, followed by *de novo* assembly of the AC sequences. The *C. elegans* genome has recently been re-sequenced multiple times and polished by long-read sequencing (49,50). Tyson *et al.* have reported the genome of a *C. elegans* strain with a biolistic-mediated insertion of a multi-copy plasmid, at a single site in tandem copies, which occupies about 2 Mbs (49). In this study, we have reported the *de novo* assembly of the genome of a *C. elegans* strain carrying an independently maintained AC, with a size of more than 10 Mbs.

Injected supercoiled DNA can form high molecular weight concatemers formed by homologous recombination, while injection of linear DNA can form HMW non-tandem ACs by random end-joining (10). Our previous study has shown that inhibition of homologous recombination (HR, by RAD-51 knockdown) or non-homologous end-joining (NHEJ, by LIG-4 knockdown) reduce AC number in embryos, indicating that both HR and NHEJ are involved in AC formation (8). Here, we also found that *lig-4* RNAi leads to formation of smaller ACs (Supplementary Figure S5D-E). Tiny ACs generated from microinjection of short DNA can barely segregate during mitosis, even in late embryonic stage (Supplementary Figure S5A), which is consistent with another study of us that shows how AC size affects the it acquisition of segregation competency (11). We reported the sequence structure of a propagated AC formed by co-injection of a high concentration of enzyme-digested blunt-ended yeast genomic DNA and a low concentration of a selectable marker and 2 differentially expressed marker genes (NGM), in a ratio of 300:1. We found that that injected DNA fragments that are incorporated to form AC are length-biased, where small injected DNA (<500 bp) is inefficient to be incorporated into the ACs (Figure 3C and Supplementary Figure S5D-E). This is possibly because the shorter the injected DNA, the more repairing processes are needed.

Based on the alignment of sequences of AC contigs to the enzyme digested yeast fragments, we propose that non-homologous end-joining is the dominant pathway to fuse small exogenous DNA fragments (mainly 500 bp–2 kb) to form a HMW DNA array (>10 Mb) (Figure 3D and Supplementary Figure S5B). Inhibiting the NHEJ pathway by depleting *lig-4* shows a significant reduction of AC size and number per cell in one-cell embryos (Supplementary Figure S5D). Double strand breaks (DSBs) adjacent to a region of homology can stimulate homologous recombination within 25 kb in *C. elegans* (51). Although there are two *unc-54* 3′ UTR regions on a NGM marker, which are sufficient to mediate homologous recombination (HR), we have not identified any recombined sequences from the two NGM markers (Supplementary Table S8 and Supplementary Figure S5C). Instead, full-length NGM marker or its partial fragments are individually interspaced by yeast genomic DNA (Figure 3D), consistent with having NHEJ dominating the fusion of microinjected foreign DNA fragments in the *C. elegans* gonad. It is possible that NHEJ outcompetes HR when a large number of DNA fragments without homologous regions are present, with only a low abundance of DNA fragments with HR regions. HR efficiency and usage may depend on the speed of pairing up with homologous sequences. Indeed, the inhibition of NHEJ factor increases the use of HR in CRISPR-induced double-strand break repairing in *C. elegans* (52).

As AC formation and *de novo* centromere formation on ACs in *C. elegans* is robust, it is interesting to know the localization of centromeres on the propagated AC. We examined the CENP-A^HCP-3^ localization on this propagated AC by ChIP-seq after we assembled the AC’s sequences. We also compared the sequence pattern of CENP-A^HCP-3^ domain between endogenous chromosomes and the propagated AC. We found a weak correlation of AT% with CENP-A^HCP-3^ domain sizes in endogenous chromosomes and AC (Figure 4G). This phenomenon may suggest that AT-rich sequences are preferred for more stable maintenance of centromeres and holocentromeres (39,40). However, the functional significance of CENP-A^HCP-3^ domain size is still unclear, as we do not know whether only larger CENP-A^HCP-3^ domains are used to form the kinetochore and attach to microtubules during mitosis. Two yeast centromeric and mitochondrial DNA sequences, which have about >80% AT, are CENP-A^HCP-3^-negative (Figure 5A). Indeed, the AT-content CENP-A^HCP-3^ domains in endogenous chromosomes or AC are all lower than 80% (Figure 4G). Therefore, CENP-A^HCP-3^ in *C. elegans* may not able to stay on extremely high AT regions.

Our previous study using fixed ChIP-chip to identify CENP-A^HCP-3^ position has found that CENP-A^HCP-3^ domains in *C. elegans* occupy about 50% of the genome, however, the amount of CENP-A^HCP-3^ protein can only occupy about 4% of the genome (18). This discrepancy could be because the ChIP samples were from a pool of millions of embryos, and the ChIP-chip analysis is showing an average in the population. Coincidently, in human centromere, CENP-A nucleosomes also occupy about 4% of the centromeric chromatin (53). In this study by fixed ChIP-seq, we found that the CENP-A^HCP-3^ domains/peaks occupy about 25.39% of the *C. elegans* genome and 17.62% of the AC sequence. The lower percentage found in our study as compared to our previous fixed ChIP-chip is possibly due to the higher resolution of ChIP-seq than ChIP-chip, and we did not merge domains with small gaps. To further investigate the position of CENP- A^HCP-3^ nucleosomes based on our ChIP-seq data, we used fragment density V-plots (54) from germline ATAC-seq (public data from Serizay *et al.* (55)) to visualize the distribution of fragment lengths relative to the distance to the center of CENP-A^HCP-3^ domains/peaks. We found that a high density of ∼200-bp fragments flank the center of CENP-A^HCP-3^ peaks that are <500 bp. This finding suggests that di-CENP-A^HCP-3^ nucleosomes might occupy at those positions (Supplementary Figure S8A). On the other hand, in a CENP-A^HCP-3^ MNase ChIP-seq study, CENP-A^HCP-3^ domains identified by the previous ChIP-chip can be consistently found (18). However, about 700 peaks of CENP-A^HCP-3^ of single nucleosome size were also found, which occupy only about 0.069% of the genome. These peaks may represent common positions among cell population (56).

The majority of CENP- A^HCP-3^ on the AC are in dispersed peaks, suggesting single CENP-A^HCP-3^ nucleosomes are interspersed between H3 nucleosomes (Figure 4I, right panel). This may explain the lower percentage of CENP- A^HCP-3^ occupancy found on the AC than that on endogenous chromosomes. As the AC consists of mostly fragmented yeast genomic DNA, and there are not many intact genes, thus CENP-A^HCP-3^ is permissible in many locations throughout the AC.

**Figure 6.**
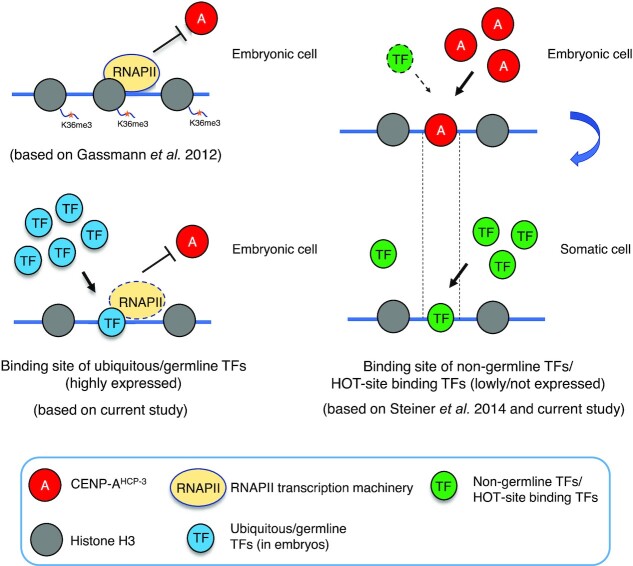
A schematic diagram of the holocentromere localization in *C. elegans* embryos. CENP-A^HCP-3^ occupancy has an inverse correlation with germline transcription in endogenous chromatin (based on Gassmann *et al.*, 2012 (18), shown on the left top); CENP-A^HCP-3^ colocalizes with transcription HOT sites, where TFs occupy the centromeric regions after cells exit mitosis or without CENP-A^HCP-3^ expression, for maintaining the centromere positions (based on Steiner *et al.* (56), shown on the right); CENP-A^HCP-3^ was excluded from transcription active regions that are preferably bound by TFs that are highly expressed in germline, but CENP-A^HCP-3^ can occupy the transcriptionally inactive regions that are preferably bound by non-germline expressed TFs (based on current study).

The centromeres in most organisms are located in intergenic regions. Integrating an activated gene into the centromere of *Candida albicans* (57) or tethering transcription activators to the human artificial chromosome (HAC) centromeric chromatin can disrupt centromere function (58). Previous ChIP-microarray results showed that CENP-A^HCP-3^-enriched regions in *C. elegans* embryos were excluded from the embryonic or germline transcribed regions (18). Ectopic expression of certain somatic genes in the *met-1* mutant germline resulted in the exclusion of CENP-A^HCP-3^ in those areas (18). These findings suggest that non-expressed regions are the preferred sites for holocentromere localization and maintenance in *C. elegans*, whereas the expressed regions or germline-expressed regions may contain memory markers that inhibit CENP-A^HCP-3^ deposition (Figure 6) (18). To test if this rule in endogenous holocentromeres also applies to the *de novo* holocentromere formed and propagated on ACs, we constructed plasmids that contain reporter genes under different stage-specific promoters and co-injected them with p64xlacO for analyzing the AC segregation efficiency. However, we cannot observe the germline expression of GFP driven by a ubiquitous *his-72* promoter, or germline *mex-5* or *pie-1* promoter (data not shown). Indeed, transgenes, especially those on repetitive ACs, were commonly suppressed in *C. elegans* germline by chromatin remodeling factors and RNAi factors (59). Though our AC is rather complex, it still contains multiple copies (>10) of the marker genes (Supplementary Table S9), thus it is not surprising that they were silenced. We have shown that CENP-A^HCP-3^ signal is negative on the ubiquitous marker gene but positive on the somatic gene and partially positive on the silenced germline gene. These results suggest that CENP-A^HCP-3^ could be enriched on silenced germline genes, but the CENP-A^HCP-3^ expansion may be inhibited by the nearby transcriptionally active region (Figure 5B). Besides, the CENP-A^HCP-3^ pattern on coding region is not directly related to its downstream 3′ UTR, since different genes with the same 3′ UTR could have different CENP-A^HCP-3^ patterns on the coding sequences (Supplementary Figure S7B). Therefore, we conclude that CENP-A^HCP-3^ on *de novo* holocentromere can also be excluded by ubiquitous transcription, and the unexpressed gene regions are permissive for *de novo* holocentromere localization. The fragmented yeast sequences do not contain many intact yeast genes, and whether any of the yeast genes are expressed in worms is currently unknown.

The most abundant motif we found from the CENP-A^HCP-3^-enriched AC domains is a 29-bp adenine-rich sequence (Figure 5C). We also identified a similar adenine-rich motif from the endogenous CENP-A^HCP-3^ peaks that are less than 500 bp (Supplementary Figure S7C). Interestingly, by comparing with the protein motif database, several transcription factor motifs, like HLH-4, HLH-15, HLH-14, BLMP-1 and EOR-1 have high similarity with the adenine-rich CENP-A^HCP-3^ motif found in the AC, and some of these TF motifs also show similarity with the adenine-rich CENP-A^HCP-3^ motif found in endogenous chromosomes (Figure 5C, Supplementary Figure S7C, and D). And these transcription factors are somatic. Chromatin accessibility, as indicated by the ATAC-seq peaks, is correlated with gene expression. An ATAC-seq study has shown a dynamic change in nucleosome accessibility in chromatin between different development stages of *C. elegans* (60). Specific binding sites of BLMP-1 and EOR-1 were predicted to be more enriched with ATAC-seq peaks in L3 stage than in early embryos (60), indicating that their expression is relatively low in embryos.

A previous study have found that transcription factors (TFs) high-occupancy target (HOT) sites colocalize with native ChIP CENP-A^HCP-3^ enrichment peaks on endogenous chromosomes, and suggested that TFs occupy CENP-A^HCP-3^ regions for maintaining the centromere position after cells exit mitosis when there is no CENP-A^HCP-3^ expression (56) (Figure 6). One of the CENP-A^HCP-3^ motifs (56) found in the CENP- A^HCP-3^ native-ChIP study is identical to the EOR-1-binding motif (61) also has sequence similarity to our A-rich motifs in endogenous and AC centromere (Supplementary Figure S7C and S7D). Taking into consideration that the chromatin status of HOT sites are dynamic in different development stages of *C. elegans* (62), CENP-A^HCP-3^ in embryos seem to prefer to localize to somatic gene regions, which are potentially bound by somatic transcription factors later on in development. Indeed, for endogenous chromosomes, CENP-A^HCP-3^ is mostly excluded from the regions with early embryo- or germline-expressed genes, but partially overlaps with somatic genes (Supplementary Figure S8B). A recent study has identified motifs enriched in the promoter regions of tissue-specific genes (55). We found that the motifs enriched in the somatic gene promoters have high sequence similarity to the CENP-A^HCP-3^ motif in our propagated AC (55) (Figure 5C). However, in the CENP-A^HCP-3^-negative regions of the propagated AC, we have not found any periodic WW-motif that is enriched in germline-specific gene promoters (55).

Our findings indicate that sequences commonly bound by somatic TFs, which could be transcriptionally inactive in embryos (Figure 6 and Supplementary Figure S8C), are potentially preferred for CENP-A^HCP-3^ maintenance. In contrast to the somatic gene regions, germline and ubiquitous genes which are activated by germline-TFs, repels CENP-A^HCP-3^ deposition (Figure 6 and Supplementary Figure S8C).

Overall, this holocentric model organism system, amenable to foreign DNA injection and AC formation with *de novo* holocentromere formation, allows us to manipulate the input DNA, compare the *de novo* centromere formation efficiency by live-cell imaging, and analyze the positions of CENP-A^HCP-3^ domains in the propagated AC. We have compared several DNA sequence parameters in order to elucidate the preferences and their potential functions in *de novo* holocentromere formation in this *in vivo*, real-time system and in holocentromere maintenance through generations. Our findings of *de novo* centromere localization in a propagated, complex AC are consistent with holocentromere features of endogenous *C. elegans* chromosomes. These results validate the use of *de novo* centromere formation on ACs to simulate centromere formation of endogenous chromosomes, potentially including events that occur during evolution and in neocentromere formation in pathological conditions.

## DATA AVAILABILITY

Whole genome sequencing data from this study have been submitted to NCBI BioProject database (BioProject; https://www.ncbi.nlm.nih.gov/bioproject/) under accession numbers PRJNA704323. ChIP-seq data from this study have been submitted to have been submitted to GEO database under accession numbers GSE145600.

## Supplementary Material

gkab690_Supplemental_Files
