# Supplementary material for: Formation of artificial chromosomes in Caenorhabditis elegans and analyses of their segregation in mitosis, DNA sequence composition and holocentromere organization
Source: Nucleic Acids Res. 2021 Aug 20;49(16):9174–93. doi: 10.1093/nar/gkab690 (PMC8450109; doi:10.1093/nar/gkab690)
Supplement: gkab690_Supplemental_Files [file gkab690_supplemental_files.zip › ACdenovo_Supplementary_Table&FigureLegends_20210722.pdf]

SUPPLEMENTARY TABLE LEGENDS AND FIGURE LEGENDS

Table S1. Sequence information of the synthetic DNA fragments with different AT-contents. For each of the 5 different AT-contents (26%, 38%, 50%, 62% and 74%), 10 1.2-kb random sequences, with a LacI binding site (LacO) at each end, were mixed and injected together. The 10 random DNA sequences with different AT-contents were generated by a JavaScript (Random DNA Generator). The sequences were synthesized *in vitro* by Genescript.

Table S2. Worm strains used in this study. The genotypes and reference sources are shown.

Table S3. List of primers used in this study. Their applications are shown.

Table S4. The transmission rates of AC to the next generation in L2.1 sublines without drug selection. Individual young adult worms from strain L2.1 were placed on EZ plates, seeded with OP50, without adding G418 antibiotic, and allowed to lay eggs for 48 hours. Progenies carrying ACs were identified by the positive mCherry fluorescence from their body wall muscles.

Table S5. A summary of MinION sequencing data from two flow-cells. Pass reads are high-quality reads isolated when the Phred quality is > 6.

Table S6. A summary of the mappability of MinION All and Pass reads to the *C. elegans* reference genome (WS245).

Table S7. A summary of genome assemblies and polishing, using a *C. elegans* strain (WYY35) carrying an artificial chromosome, based on different genome assembly pipelines using MinION, Mi-seq and combined datasets.

Table S8. The locations of the three full-length marker genes and partial components in the AC.

Table S9. The copy number of the three marker gene components in the AC.

Figure S1 The relationship between injected DNA sequence characteristics and AC

segregation frequency. (A) Representative images of the cells with segregating ACs after injection of plasmid DNA with different AT-contents. The 52% AT DNA fragment was PCR amplified from the genomic DNA of budding yeast *Saccharomyces cerevisiae* using the primers AT-52-F and AT-52-R. The 66% AT DNA fragment was amplified from the genomic DNA of budding yeast using the primers AT-66-F and AT-66-R. The 74% AT DNA fragment was amplified from the mitochondrial DNA of budding yeast using the primers AT-74-F and AT-74-R. The amplified DNA fragments (~5 kb) with different AT-contents were cloned into plasmid pMD-19T (2694 bp) by TA cloning to make plasmids pAT-52%, pAT-64% and pAT-74% (~7.8 kb in total), respectively. Circular pAT-52%, pAT-66% or pAT-74% was co-injected with the p64xLacO plasmid in a ratio of 10:1, at 91 ng/μl and 9 ng/μl, respectively, to generate ACs with different AT-contents. All types of DNA for microinjection were adjusted to 100 ng/μL in total. The percentage of cells with segregating ACs among all dividing cells containing ACs was scored. (B) Quantification of the cells with segregating ACs, among all dividing cells containing ACs, after injection of plasmid DNA with different AT-contents. The number of cells (n) analyzed was indicated. Blue arcs show comparisons among pAT-52%, pAT-66% and pAT-74% at each cell stage. In 1-4-, 5-8- and 9-16-cell stage embryos, the segregation rates of ACs generated from pAT-52% and pAT-66% show no significant difference. However, the segregation rates of ACs made with pAT-74% are significantly higher than those of pAT-52% and pAT-66%. At 17-32-cell stages, the segregation rates of ACs generated from the three AT-contents become similar. The segregation competency of all types of ACs improves over time. The difference of their segregation competency is more significant in early-stage embryos than in late-stage embryos. (C) ACs with repetitive structure were generated by injecting just the linear plasmid (L64xLacO) (same data are used for (1) Figure S1C). ACs with a complex sequence context were generated by injection of a mixture of linear plasmid (L64xLacO) and sheared salmon sperm DNA (SS-DNA) (~5 kb) in a concentration ratio of 1 L64xLacO:10 SS-DNA. The AT% of L64xLacO is 52%, the AT% of salmon sperm DNA is 58.8%. Both complex and repetitive types of DNA for microinjection were adjusted to a final concentration of 100 ng/μL. 5 hours after injection of L64xLacO or L64xLacO with SS-DNA, representative embryos expressing GFP::lacI (green) and mCherry::H2B (red) and carrying multiple ACs are shown by live-cell time-lapse imaging. Yellow arrowheads point to the AC undergoing segregation from 1-cell to 4-cell stage. The time (mm:ss) is indicated on the top right of images. These ACs mature from passive inheritance to autonomous segregation rapidly within a few cell cycles. Scale bar represents 5 μm. (D) Quantification of the percentage of

cells with segregating ACs, among all dividing cells containing ACs, after injection of DNA with different sequence complexity. The number of cells (n) analyzed was indicated. Fisher's exact test was used to test for significance. NS means not significant.

Figure S2. (A) Budding yeast *Sacchromyces cerevisiae* genomic DNA was digested with restriction enzymes *Afa*I (GT|AC) and *Pvu*II (CAG|CTG). A histogram of *in silico* digestion is shown. (B) Genomic DNA was isolated from an S288C background strain, BY4742. An agarose gel image showing the sizes of enzyme-digested DNA (*Afa*I and *Pvu*II). 150 ng/µl of this digested DNA was used for microinjection. (C) A representative image that shows an AC in an oocyte (Green, Alexa 488 FISH probes made from yeast genomic DNA). Scale bar represents 5 µm. The integrated density of DAPI on the 6 diakinesis endogenous chromosomes (4C) corresponds to 400 Mb. The integrated density of DAPI on the propagated AC, assumingly replicated and in monosomy, was used to calculate the size of this AC. ACs and endogenous chromosomes from 9 individual oocytes were used for quantification. The bar chart shows the AC size (2C) that is normalized to endogenous chromosomes. The error bar indicates standard deviation (SD). The AC size (1C) was estimated to be around 13 Mb. (D) A representative image shows that a propagated AC that lacks AIR-2 signal in an oocyte (Red, AIR-2; Green, yeast genomic DNA probes; Blue, DAPI). Scale bar represents 5 µm. (E) A representative image shows that CENP-$A^{HCP\text{-}3}$ staining of a propagated AC in an oocyte. The bar chart shows the quantification of normalized CENP-$A^{HCP\text{-}3}$ integrated density on the AC in oocytes. (F) A representative image shows that CENP-$A^{HCP\text{-}3}$ staining of a propagated AC in an embryo (Red, CENP-$A^{HCP\text{-}3}$; Green, yeast genomic DNA probes; Blue, DAPI). Scale bar represents 5 µm.

Figure S3. Whole-genome sequencing of a *C. elegans* strain with a complex artificial chromosome L2.1 (WYY35). Raw data produced from MinION flow-cells were base-called by Metrichor. (A, B) Histograms of the read numbers were plotted against the read length yielded from each flow-cell (A: FAF03915; B: FAF03410). The blue line indicates the N50 of read length. (C, D) Histograms of read length and Phred quality in each flow-cell (C: FAF03915; D: FAF03410). The number of reads from each flow-cell in different read lengths and read qualities were indicated by colors. (E) MinION individual read accuracies and read coverage when aligned to the reference worm genome (WS245). Graphmap aligner was used for mapping. Read base-called qualities were plotted against alignment read percentage identity.

Most of the reads have a percentage identity of about 85-90%, but with a long tail to identities of ~60%. Pearson correlation: r = 0.75, p-value = 0. A dashed red oval shows a small portion of high-quality reads with low mapping identity, indicating these reads might not belong to the worm genomic DNA. (F) Alignment percentage identity was plotted against aligned read length. This is a comparison of mapping quality for different read lengths after alignment using Graphmap aligner. No length bias was observed, with long reads having similar percentage identity as short ones. Pearson correlation: r = 0.04. (G) Coverage for *C. elegans* reference genome (WS245) based on All reads generated from 2 MinION flow-cells. (H) Coverage for *C. elegan*s reference genome (WS245) based on Pass reads, which are filtered based on Phred quality > 6.

Figure S4. (A) Mummer alignments of assembled contigs from MinION reads to *C. elegans* reference genome (WS245). The dot plot displays the one-to-one mapping between the assembled contigs sets (on the Y-axis) and the reference chromosomes (on X-axis). Forward matches are indicated in red and reverse complement matches in blue. (B) Workflow for isolating AC reads from the whole *C. elegans* genome sequencing (WGS). *S. maltophilia* sequences were found in direct whole genome assembly using All reads (“Bacteria Filter”). Bacterial contaminated reads were first removed if they aligned to the bacterial genome. Then, reads that aligned to the and NGM markers and *in silico* digested yeast genome were isolated (“NGM & Yeast Filter”) and combined with reads that did not align to the *C. elegans* genome (“Worm Genome Filter”). In “Worm Genome Filter”, reads were aligned to the *C. elegans* reference genome (which deliberately excludes the sequences of promoters and 3’ UTRs that are present in the NGM markers). The reads aligned to the *C. elegans* genome were excluded. The alignments were performed by two different algorithms in parallel, Graphmap and minimap2, to acquire as many AC reads as possible. Next, the isolated reads by both algorithms were merged, followed by removing duplicates. Read numbers generated from each step were summarized in brackets. After the Canu assembly pipeline, contigs were further aligned to the *C. elegans* reference genome and filtered out if they have > 95% identity (“Remove Worm Contigs”).

Figure S5. (A) The tiny ACs formed by microinjection of 8xLacO has no segregation competency. Linear 8xLacO (315 bp) fragments were microinjected at a concentration of 100 ng/μl. No AC segregation was observed even in the late-stage embryos. (B) A representative

image shows that the alignment of yeast genomic DNA fragments (as shown by red arrows from *Afa*I and *Pvu*II double digestion) to the assembled AC (tig8258: 1,147,835-1,455,503 bp). The unfilled part of the arrow represents that this part is missing in the assembled AC. The vertical bars indicate single base pair variants. (C) Alignment of tig 8258 to the co-injection markers NGM (*Prps-27::NeoR::unc-54 3' UTR*; *Pmex5::gfp::tbb-2 3' UTR*; *Pmyo-3::mCherry:: unc-54 3' UTR*) shows that fragments from NGM were interspaced in between the yeast genomic DNA in forward and reverse directions (shown in green and red, respectively). (D) Representative images of nascent ACs generated from co-injection of restriction enzyme-digested yeast genomic DNA (*Afa*I and *Pvu*II) and L64xLacO in a ratio of 10:1, at 91 ng/μl and 9 ng/μl, respectively, in WT untreated and *lig-4* RNAi-treated one-cell embryos. Yellow arrowheads indicate the ACs. Scale bar represents 5 μm. (E) Quantification of the diameter of ACs in WT and *lig-4* RNAi-treated one-cell embryos. The numbers of embryos and ACs (n) analyzed are shown. Student's t-test was used to test the significance. ****: $p < 0.0001$.

Figure S6. The CENP-A^{HCP-3} signals around domain/peak centers for endogenous chromosomes and the AC. The profile plots (upper panel) show the average ChIP signals of each cluster. The ChIP signals of each domain/peak are shown in the clustered heatmaps (lower panel). The color coding on the right of the heatmap indicates the $\log_2$ ratio of CENP-A^{HCP-3}.

Figure S7. (A) Relative transcription level of each NGM marker gene in early embryos (normalized to their copy number). (B) Comparison of CENP-A^{HCP-3} distribution on the same 3' UTRs in the AC and endogenous chromosomes. The $\log_2$ CENP-A^{HCP-3} ratio on marker genes is averaged from 10-29 copies (Table S9). Non-distinguishable reads that mapped to both endogenous and AC promoter or 3' UTR regions were excluded from the analysis and the region with all reads removed was shaded. (C) Two motifs found in the JASPAR database, and a CENP-A^{HCP-3}-enriched motif found among CENP-A^{HCP-3} peaks (that are < 500 bps) on endogenous chromosomes show a significant match to the CENP-A^{HCP-3} motif on AC (as shown in Figure 5C). The p-value Indicates the significance of the similarity between CENP-A^{HCP-3} motif and TF motif (also for D and F). (D) Three motifs found in the JASPAR database showed a significant match to motif enriched in endogenous CENP-A^{HCP-3} peaks (< 500 bp). (E) Two motifs identified from the CENP-A^{HCP-3}-negative regions in the propagated

AC have sequence similarities with HLH-2 motifs. Toggle error bars indicate the confidence of a motif based on the number of sites used in its creation.

Figure S8. (A) V-plots of germline ATAC-seq fragment density flanking the center of ubiquitous promoters, random sequences, all CENP-A^{HCP-3} domains/peaks, CENP-A^{HCP-3} domains/peaks that are < 2000 bp, < 500 bp and < 200 bp, respectively. (B) Representative enrichment of CENP-A^{HCP-3} signal, CENP-A^{HCP-3} domains, one-cell embryo-expressed genes, data from Tintori *et al*., 2016 (2), germline-expressed genes (including germline-specific genes (blue), ubiquitous uniform (< 3-fold difference between any two tissues) (red) and ubiquitous biased (black)), non-germline-expressed genes (including neuron- (green), muscle- (orange), hypodermis- (grey), intestine- (pink) and soma-specific genes (turquoise)) on chromosome II (7.3-7.7 Mb). The ubiquitous and tissue-specific gene lists are from Serizay *et al*., 2020 (3). (C) The transcriptional landscapes of germline-expressed genes and non-germline-expressed genes. The relative expression level of germline-expressed genes and non-germline-expressed genes were compared among different developmental stages (left panels) and different tissues (right panels). The germline-expressed genes are also highly expressed in embryos, as compared with non-germline-expressed genes. The gene lists are based on Serizay *et al.*, 2020 (3); expression data are from *Janes et al, 2018* (4); heatmaps were generated by RegAtlas.

References

1. Lin, Z. and Yuen, K.W.Y. (2020) DNA Sequence Preference for De Novo Centromere Formation on a Caenorhabditis elegans Artificial Chromosome. *bioRxiv doi:10.1101/2020.04.12.037994*.
2. Sophia, Erin, Golden, P., Jason and Goldstein, B. (2016) A Transcriptional Lineage of the Early C. elegans Embryo. *Developmental cell*, **38**, 430-444.
3. Serizay, J., Dong, Y., Janes, J., Chesney, M., Cerrato, C. and Ahringer, J. (2020) Distinctive regulatory architectures of germline-active and somatic genes in C. elegans. *Genome research*, gr.265934.265120.
4. Jänes, J., Dong, Y., Schoof, M., Serizay, J., Appert, A., Cerrato, C., Woodbury, C., Chen, R., Gemma, C., Huang, N. *et al.* (2018) Chromatin accessibility dynamics across C. elegans development and ageing. *eLife*, **7**.
