# Supplementary material for: Formation of artificial chromosomes in Caenorhabditis elegans and analyses of their segregation in mitosis, DNA sequence composition and holocentromere organization
Source: Nucleic Acids Res. 2021 Aug 20;49(16):9174–93. doi: 10.1093/nar/gkab690 (PMC8450109; doi:10.1093/nar/gkab690)
Supplement: gkab690_Supplemental_Files [file gkab690_supplemental_files.zip › Table S2-7&9_Figure S1-6_20210722.pdf]

Table S2. Worm strains used in this study.

Strain name	Genotype	Reference
**N2**	Wild-type	
**OD426**	*ItIs37 [pie-1p::mCherry::his-58; unc-119(+)] IV;*<br>*meIs1 [pie-1p::GFP::LacI]*	(8)
**WYY35**	*Ex [Prps-27::NeoR::unc-54 3'UTR; Pmex5::gfp::tbb-2 3'UTR; Pmyo-3::mCherry:: unc-54 3'UTR; S. cerevisiae Afa*I, *PvuII*-digested total DNA]	This study

Table S3. List of primers used in this study.

Primer name	Sequences (5' to 3')	Application
AT-52-F	ATAATCTGCGACGAAGCTATGC	Amplification of 52% AT yeast DNA fragment
AT-52-R	AAAGGAAGGCTCAATGACGAAT	
AT-66-F	AAACGACAGCGAAGATAACG	Amplification of 66% AT yeast DNA fragment
AT-66-R	TTGGGAGCCTATTGTGAAGT	
AT-74-F	GCAACTGAACTACCAGCAAG	Amplification of 74% AT yeast mitochondrial DNA fragment
AT-74-R	CCTGAATGTGCCTGAATAGA	
M13-47	CGCCAGGGTTTTCCC AGTCA CGAC	Linearization of NGM marker by PCR
RV-M	GAGCGGATAACAATTTCACA CAGG	
gDNA_mCherry_F	TTCTGCATAACAGGTCCAT	Quantification of NGM copy number in AC
gDNA_mCherry_R	TGACGCAAGACTCAAGGTA	
Yeast_rDNA_F	CTTACTCAAATCCATCCGAAGA	
Yeast_rDNA_R	ACGGACCAAGGAGTCTAACG	
Worm_ChrII_F	GCGGATTGTGAGACTATTT	
Worm_ChrII_R	CTTGATGGAACTGAAGGAA	
cDNA_GFP_F	ACTTTTCACTGGAGTTGTCCCA	Quantification of gene expression
cDNA_GFP_R	AGGGTAAGTTTTCCGTATGTTGC	
cDNA_mCherry_F	CATCCATGCCACCTGTCGAG	
cDNA_mCherry_R	TGCCAGGAGCGTACAATGTTA	
cDNA_NeoR_F	CTGCAGTTCATTCAGGGCAC	
cDNA_NeoR_R	ATGACTGGGCACAACAGACA	

Table S4. The transmission rates of AC to the next generation in L2.1 sublines without drug selection.

Strain L2.1	mCherry positive progenies	mCherry negative progenies	Positive percentage
Subline 1	167	114	59.43%
Subline 2	144	103	58.30%
Subline 3	132	81	61.97%
Average			60.60%

Table S5. A summary of MinION sequencing data from two flow-cells.

**Flow-cell ID**	**FAF03915**		**FAF03410**	
	**Total**	**Pass reads [a]**	**Total**	**Pass reads [a]**
**Read Count**	636360	483360	805812	640037
**Total Yield**	4.92 G bases	3.92 G bases	5.03 G bases	4.18 G bases
**Sequence Length - Average**	7.74 K bases	8.10 K bases	6.24 K bases	6.53 K bases
**Sequence Length - Median**	7.48 K bases	7.48 K bases	5.78 K bases	5.78 K bases
**Sequence Length - Mode**	6.82 K bases	7.70 K bases	578.00 bases	578.00 bases
**Longest Read**	131.78 K bases	93.50 K bases	149.69 K bases	121.02 K bases
**QScore – Average**	7.1	7.8	7.2	7.8
**QScore – Median**	7.5	7.9	7.6	7.9
**QScore – Mode**	8.4	8.4	8.4	8.4

[a] : Pass reads are high-quality reads isolated when the Phred quality is > 6.

Table S6. A summary of the mappability of MinION reads to the *C. elegans* reference genome (WS245).

	**All read [a] coverage and identity**	**Pass read [b] coverage and identity**
**Reference genome size (WS245)**	100,286,401	
**Number of reads**	1,442,348	1,081,581
**Mapped reads**	1,142,250 / 79.19%	957,042 / 88.49%
**Unmapped reads**	300,098 / 20.81%	124,539 / 11.51%
**Read min/max/mean length**	5 / 149,685 / 6,902.73	5 / 95,097 / 7,363.16
**Duplicated reads (estimated)**	32,252 / 2.24%	23,280 / 2.15%
**Duplication rate**	1.70%	1.52%
**Clipped reads**	875,288 / 60.68%	741,107 / 68.52%
**Mean Mapping Quality**	34.81	35.13
**Mean Coverage**	88.8253	75.8705
**Coverage Standard Deviation**	135.5784	109.2802
**General error rate**	20.73%	18.87%
**Mismatches**	1,568,580,609	1,218,756,648
**Insertions**	192,568,865	147,374,109
**Mapped reads with at least one insertion**	99.99%	100%
**Deletions**	694,098,290	553,275,323
**Mapped reads with at least one deletion**	100%	100%
**Homopolymer indels**	37.76%	39.32%

[a] : All reads are produced from two MinION nanopore flow-cells.

[b] : Pass reads are high-quality reads isolated when the Phred quality is > 6.

Table S7. A summary of genome assemblies and polishing, using a *C. elegans* strain (WYY35) carrying an artificial chromosome, based on different genome assembly pipelines using MinION, Mi-seq and combined datasets.

	**MinION Canu (All reads[a])**	**MinION Canu (Pass reads[b])**	**Mi-seq SPAdes**	**Canu (All) + Pilon (x3) [c]**
**Total Bases**	9.95 G bases	8.1G bases	5.4 G bases	9.95 G bases + 5.4 G bases
**Contigs**	241	252	38,187	241
**Total Assembly (bp)**	114,955,672 bp	115,248,067 bp	110,413,755 bp	119,160,402 bp
**N50**	1,487,696 bp	1,404,122 bp	22,768 bp	1,515,988 bp
**Longest contig**	4,707,476 bp	4,862,513 bp	188,252 bp	4,774,338 bp
**Sequence identity to reference**	95.84%	95.84%	99.94%	99.79%
**Aligned bases (REF vs. QRY)**	100139477 (99.85%) vs. 99505683 (86.46%)	100173646 (99.89%) vs. 99624555 (86.44%)	100183478 (99.90%) vs. 98876593 (91.17%)	100147804 (99.86%) vs. 103389851 (86.77%)

[a]: All reads are produced from two MinION nanopore flow-cells.
[b]: Pass reads are high-quality reads isolated when the Phred quality > 6.
[c]: Assembled contigs using All reads by Canu are polished three rounds by Pilon.

Table S9. The copy number of the three marker gene components in the AC.

Type	Query name	Length	Hits	Query cover
full length gene	*Pmex5::gfp::tbb-2 3'UTR*	2,136	10	100.00%
full length gene	*Pmyo-3::mCherry::unc-54 3'UTR*	4,197	12	99.90%
full length gene	*Prps-27::NeoR::unc-54 3'UTR*	2,288	11	99.96%
gene cassette	*Pmyo-3*	2,503	12	100.00%
gene cassette	*Pmex-5*	486	13	100.00%
gene cassette	*Prps-27*	797	15	99.87%
gene cassette	*gfp*	867	11	100.00%
gene cassette	*mCherry*	864	19	100.00%
gene cassette	*NeoR*	785	13	100.00%
gene cassette	*unc-54 3'UTR*	760	29	100.00%
gene cassette	*tbb-2 3'UTR*	332	10	100.00%

Figure S1

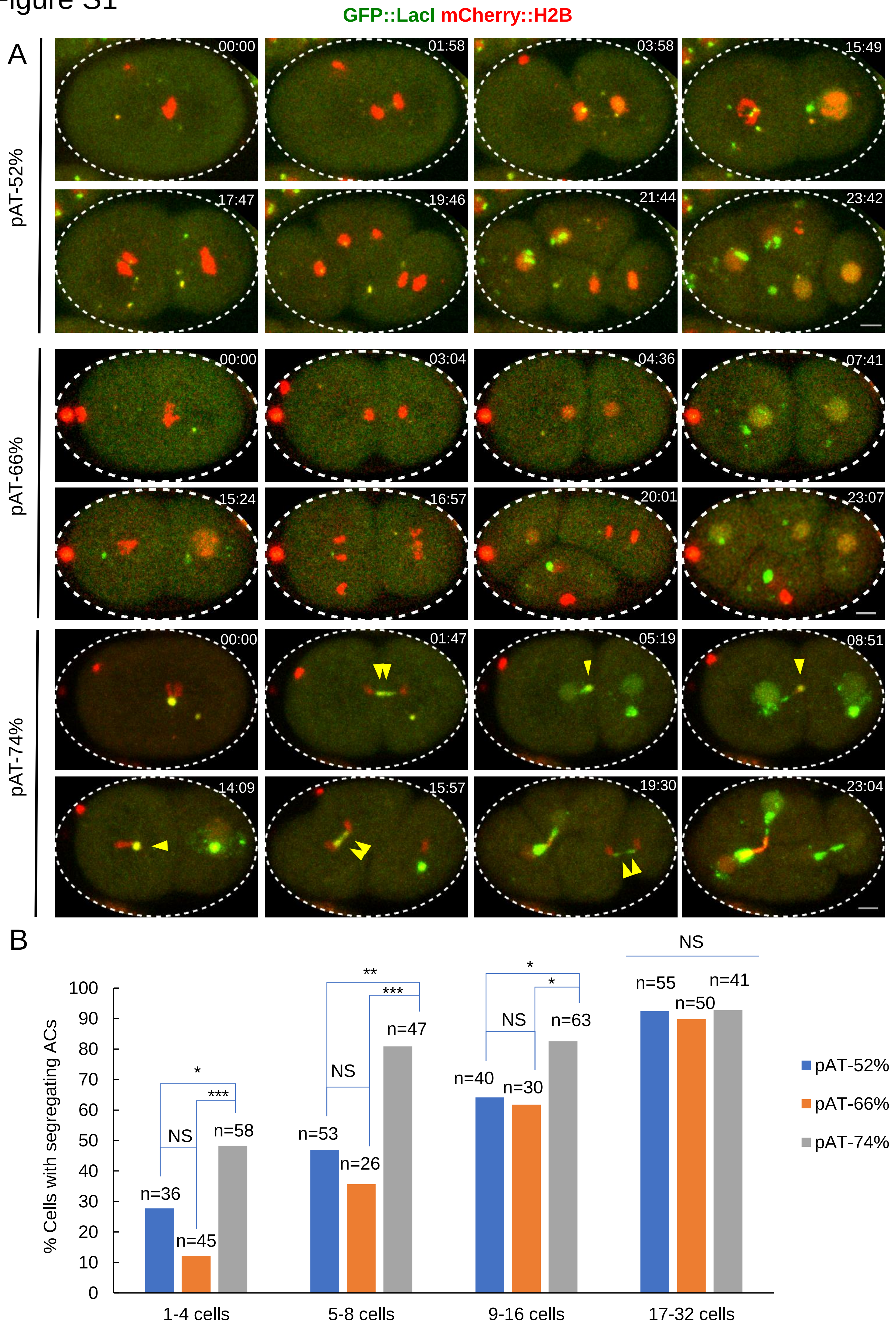
GFP::LacI mCherry::H2B
A
pAT-52%
00:00
01:58
03:58
15:49
17:47
19:46
21:44
23:42
pAT-66%
00:00
03:04
04:36
07:41
15:24
16:57
20:01
23:07
pAT-74%
00:00
01:47
05:19
08:51
14:09
15:57
19:30
23:04
B
% Cells with segregating ACs
100
90
80
70
60
50
40
30
20
10
0
1-4 cells
5-8 cells
9-16 cells
17-32 cells
n=36
n=45
n=58
NS
***
*
n=53
n=26
n=47
NS
***
**
n=40
n=30
n=63
NS
*
*
n=55
n=50
n=41
NS
pAT-52%
pAT-66%
pAT-74%

C

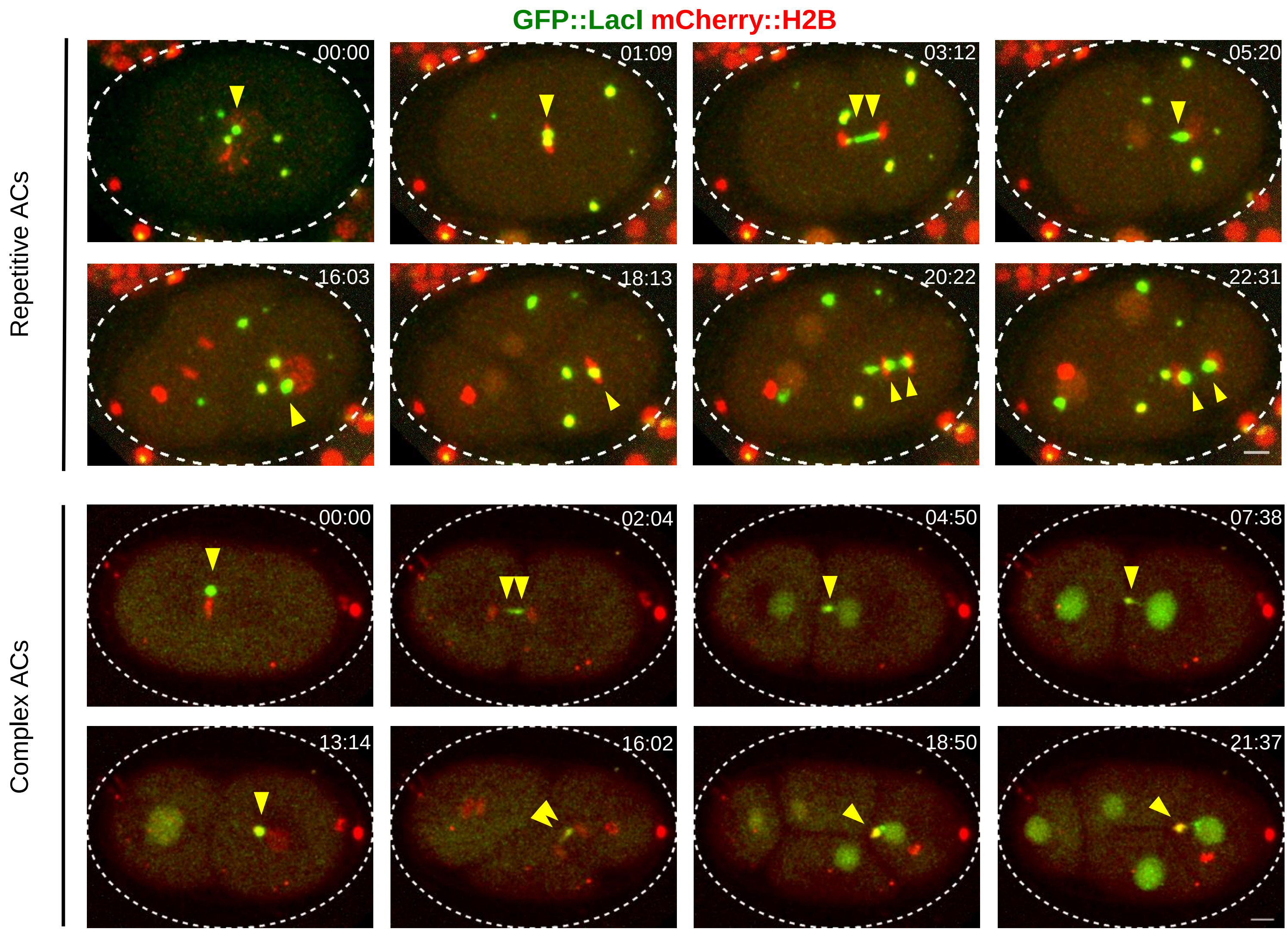
GFP::LacI mCherry::H2B
Repetitive ACs
Complex ACs
00:00
01:09
03:12
05:20
16:03
18:13
20:22
22:31
00:00
02:04
04:50
07:38
13:14
16:02
18:50
21:37

D

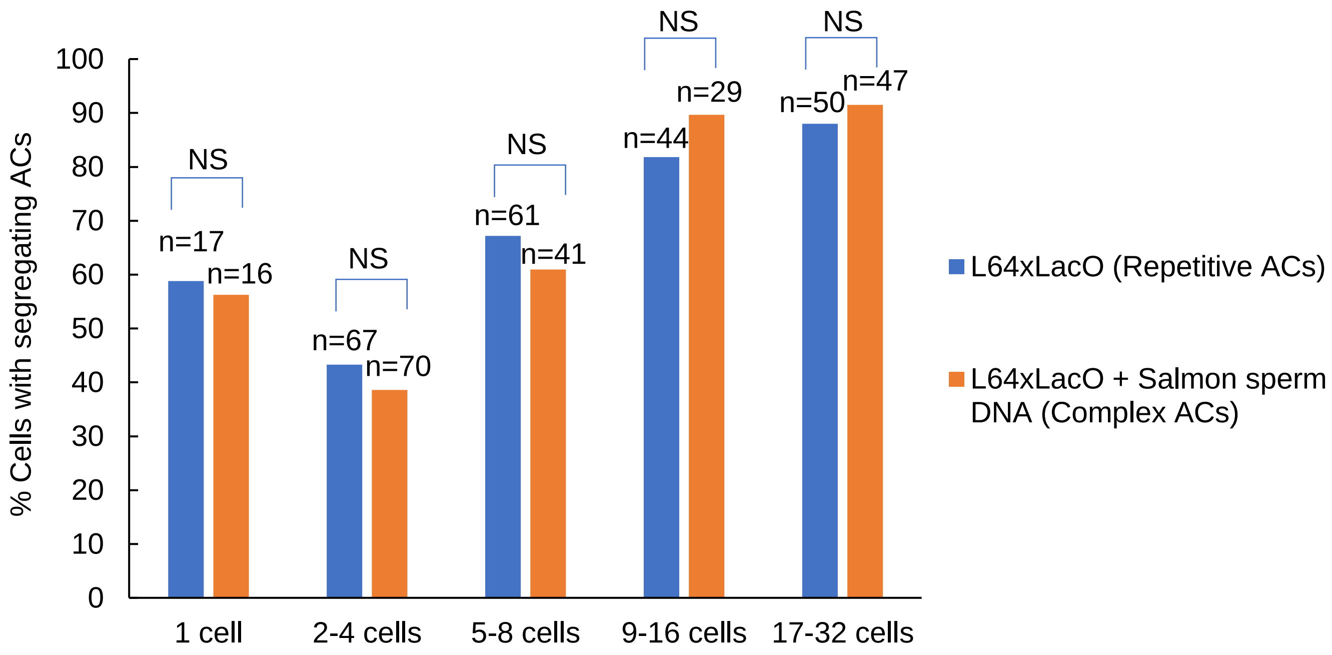
% Cells with segregating ACs
100
90
80
70
60
50
40
30
20
10
0
NS
n=17
n=16
n=67
n=70
n=61
n=41
n=44
n=29
n=50
n=47
1 cell
2-4 cells
5-8 cells
9-16 cells
17-32 cells
L64xLacO (Repetitive ACs)
L64xLacO + Salmon sperm DNA (Complex ACs)

Figure S2

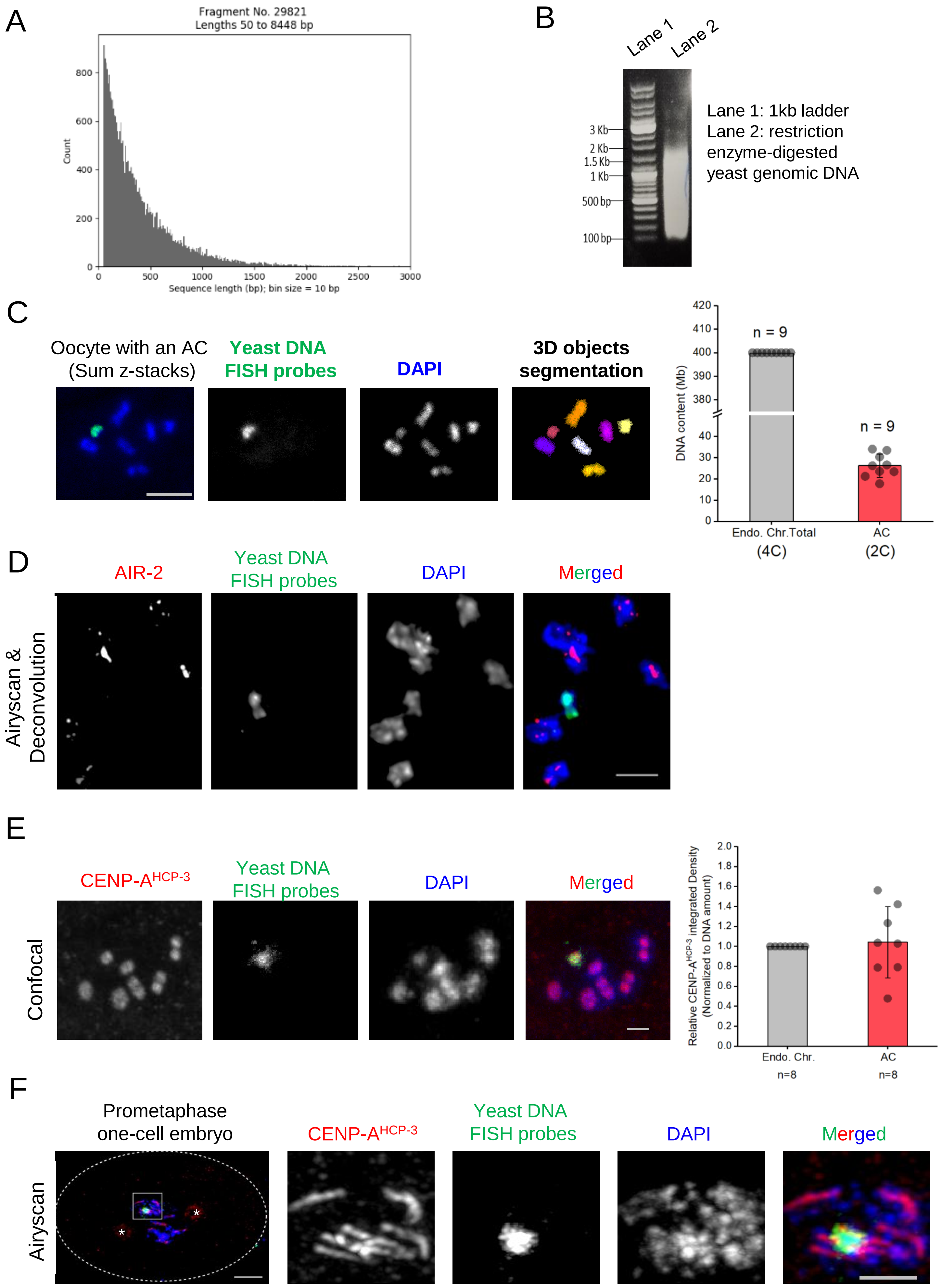

A
Fragment No. 29821
Lengths 50 to 8448 bp
Count
Sequence length (bp); bin size = 10 bp
B
Lane 1
Lane 2
3 Kb
2 Kb
1.5 Kb
1 Kb
500 bp
100 bp
Lane 1: 1kb ladder
Lane 2: restriction enzyme-digested yeast genomic DNA
C
Oocyte with an AC (Sum z-stacks)
Yeast DNA FISH probes
DAPI
3D objects segmentation
DNA content (Mb)
n = 9
n = 9
Endo. Chr.Total (4C)
AC (2C)
D
AIR-2
Yeast DNA FISH probes
DAPI
Merged
Airyscan & Deconvolution
E
CENP-A HCP-3
Yeast DNA FISH probes
DAPI
Merged
Confocal
Relative CENP-A HCP-3 integrated Density (Normalized to DNA amount)
Endo. Chr. n=8
AC n=8
F
Prometaphase one-cell embryo
CENP-A HCP-3
Yeast DNA FISH probes
DAPI
Merged
Airyscan

Figure S3

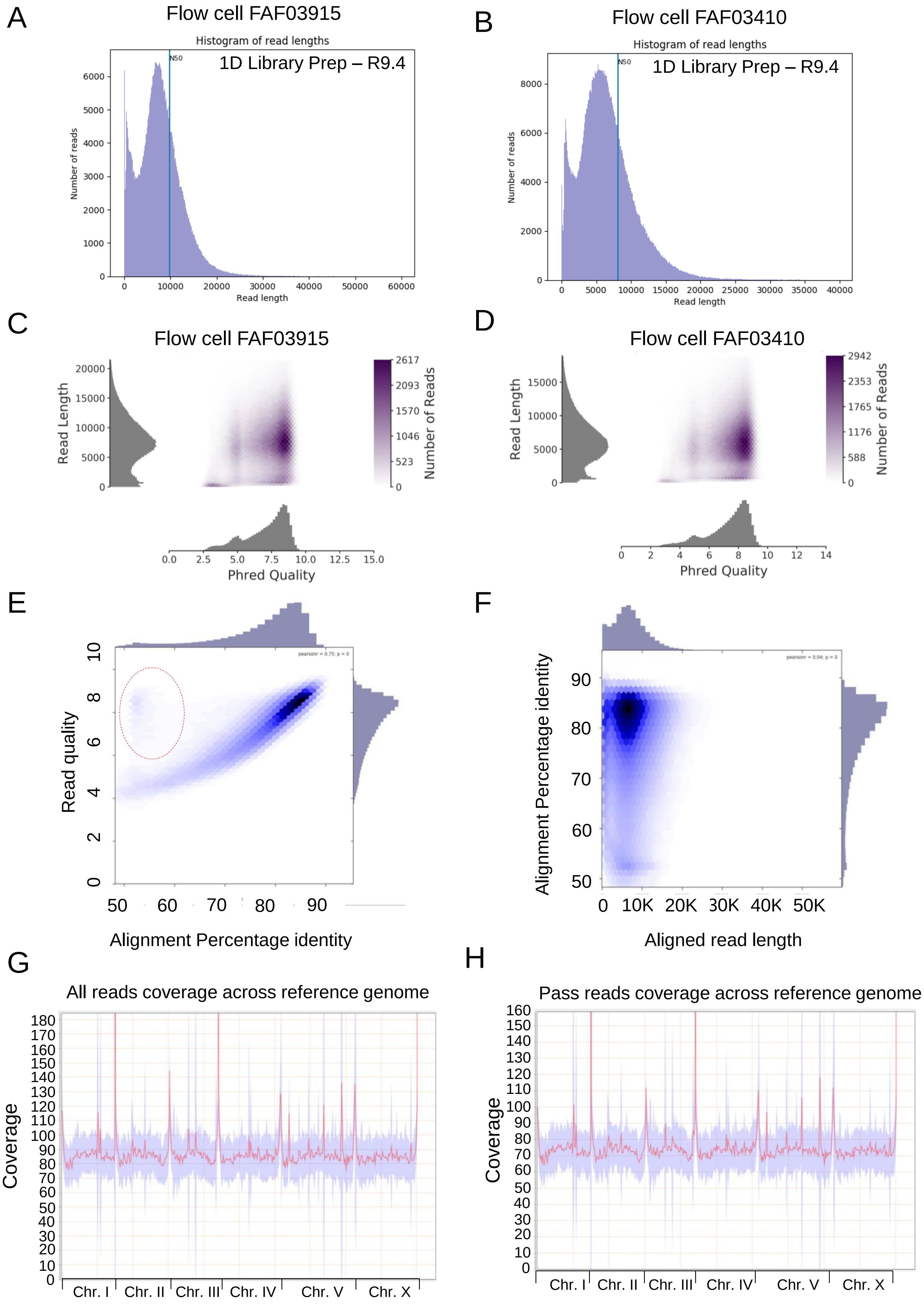
A
Flow cell FAF03915
Histogram of read lengths
1D Library Prep – R9.4
N50
Number of reads
Read length
B
Flow cell FAF03410
Histogram of read lengths
1D Library Prep – R9.4
N50
Number of reads
Read length
C
Flow cell FAF03915
Read Length
Number of Reads
Phred Quality
D
Flow cell FAF03410
Read Length
Number of Reads
Phred Quality
E
Read quality
Alignment Percentage identity
F
Alignment Percentage identity
Aligned read length
G
All reads coverage across reference genome
Coverage
Chr. I
Chr. II
Chr. III
Chr. IV
Chr. V
Chr. X
H
Pass reads coverage across reference genome
Coverage
Chr. I
Chr. II
Chr. III
Chr. IV
Chr. V
Chr. X

Figure S4

A

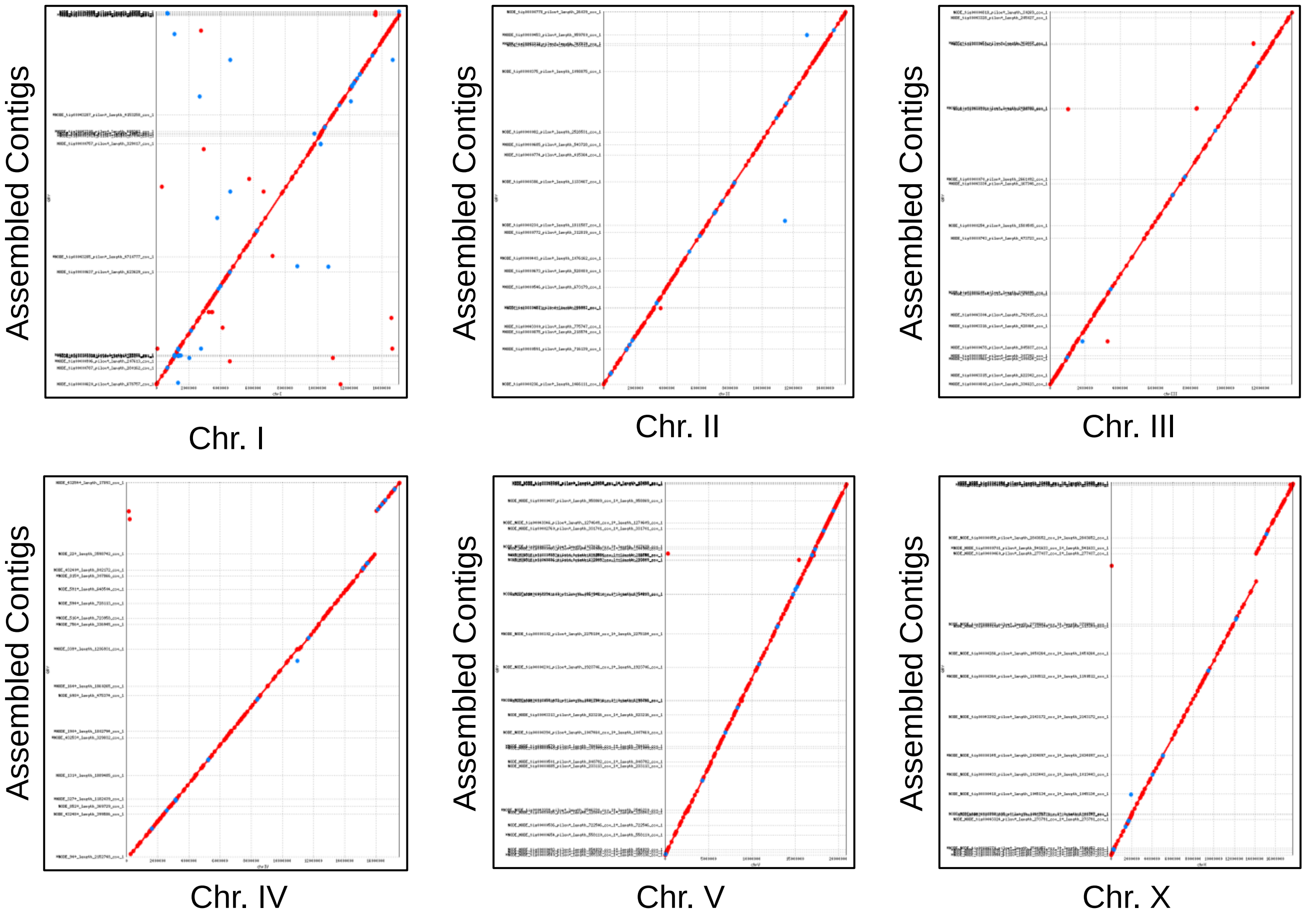

B

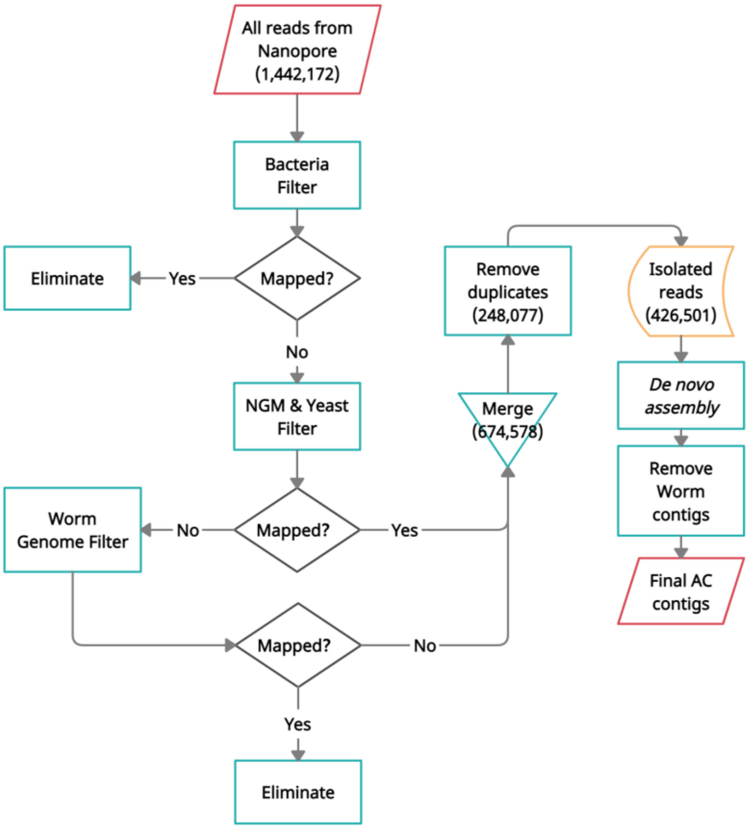

	Mapped (reads)		Unmapped (reads)	
Software	**Graphmap**	**Minimap2**	**Graphmap**	**Minimap2**
Bacteria Filter	61,246	57,287	1,381,246	1,386,827
NGM & Yeast Filter	217,970	104,003	1,163,294	1,282,824
Worm Genome Filter	1,005,587	1,087,926	157,707	194,898

Figure S5

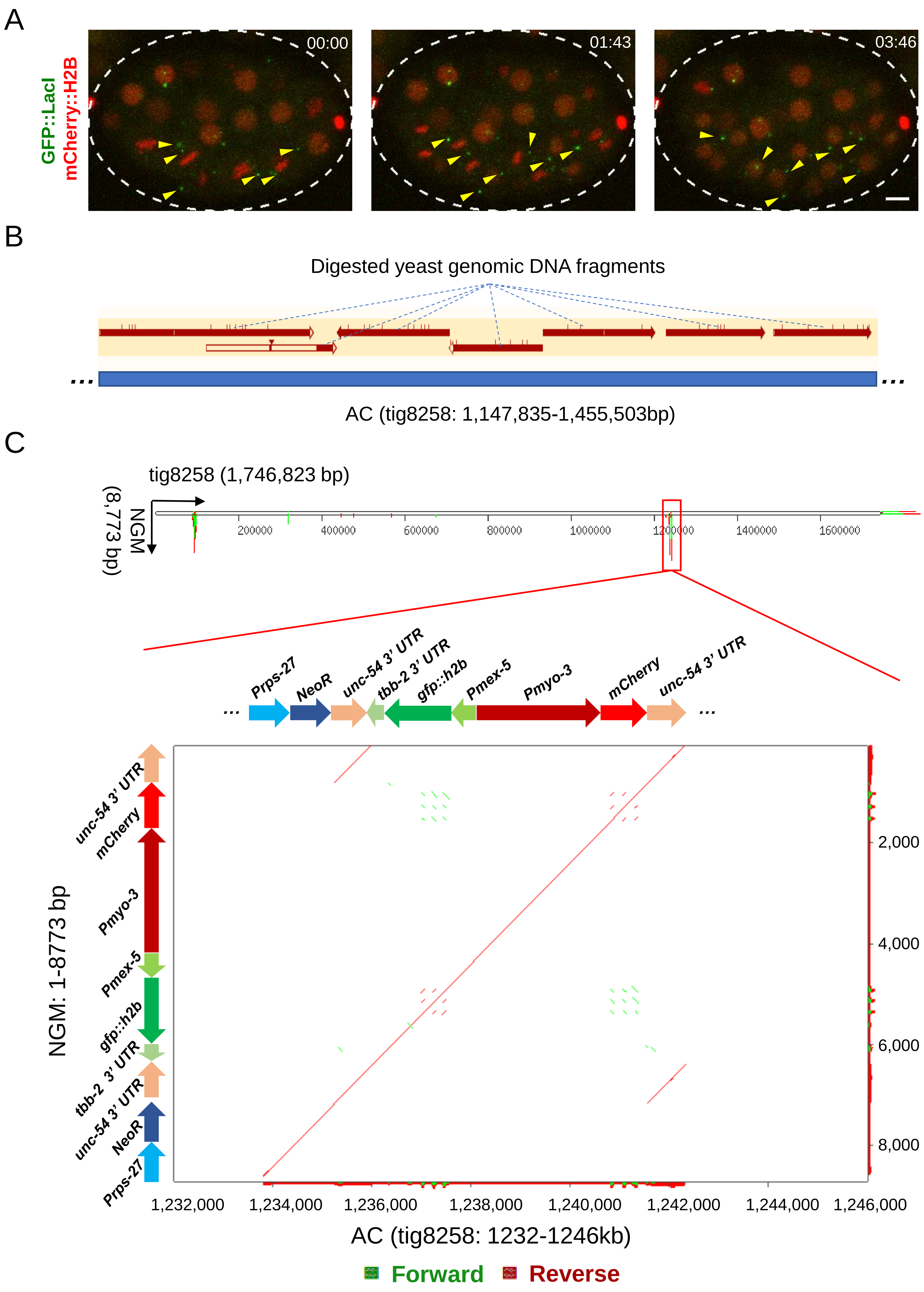
A
00:00
01:43
03:46
GFP::LacI
mCherry::H2B
B
Digested yeast genomic DNA fragments
AC (tig8258: 1,147,835-1,455,503bp)
C
tig8258 (1,746,823 bp)
NGM (8,773 bp)
200000
400000
600000
800000
1000000
1200000
1400000
1600000
Prps-27
NeoR
unc-54 3' UTR
tbb-2 3' UTR
gfp::h2b
Pmex-5
Pmyo-3
mCherry
unc-54 3' UTR
NGM: 1-8773 bp
2,000
4,000
6,000
8,000
1,232,000
1,234,000
1,236,000
1,238,000
1,240,000
1,242,000
1,244,000
1,246,000
AC (tig8258: 1232-1246kb)
Forward
Reverse

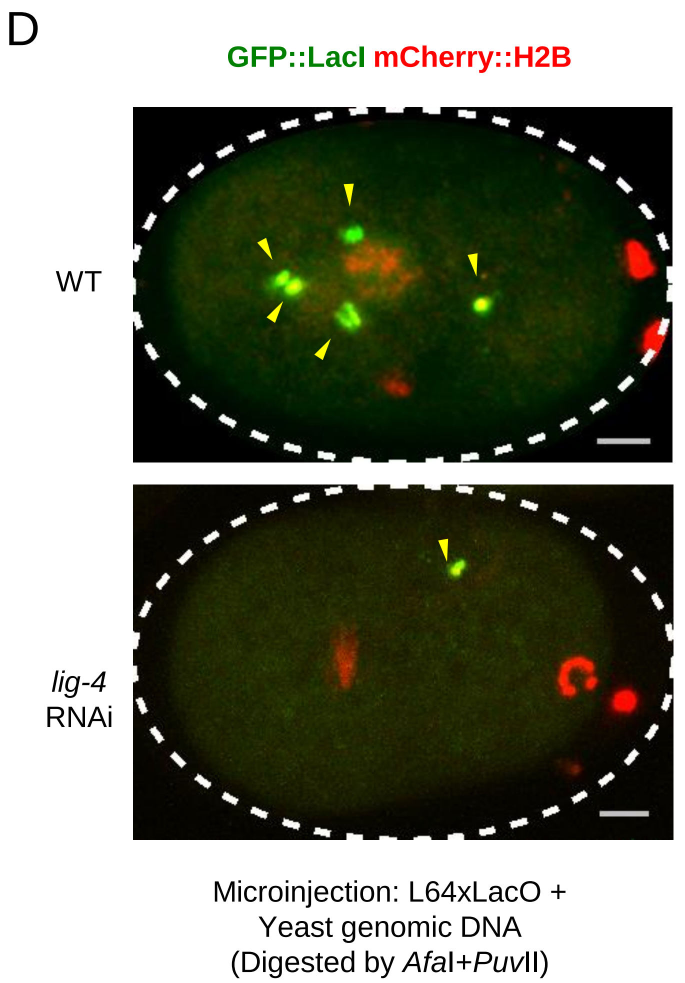
D
GFP::LacI mCherry::H2B
WT
lig-4
RNAi
Microinjection: L64xLacO +
Yeast genomic DNA
(Digested by AfaI+PuvII)

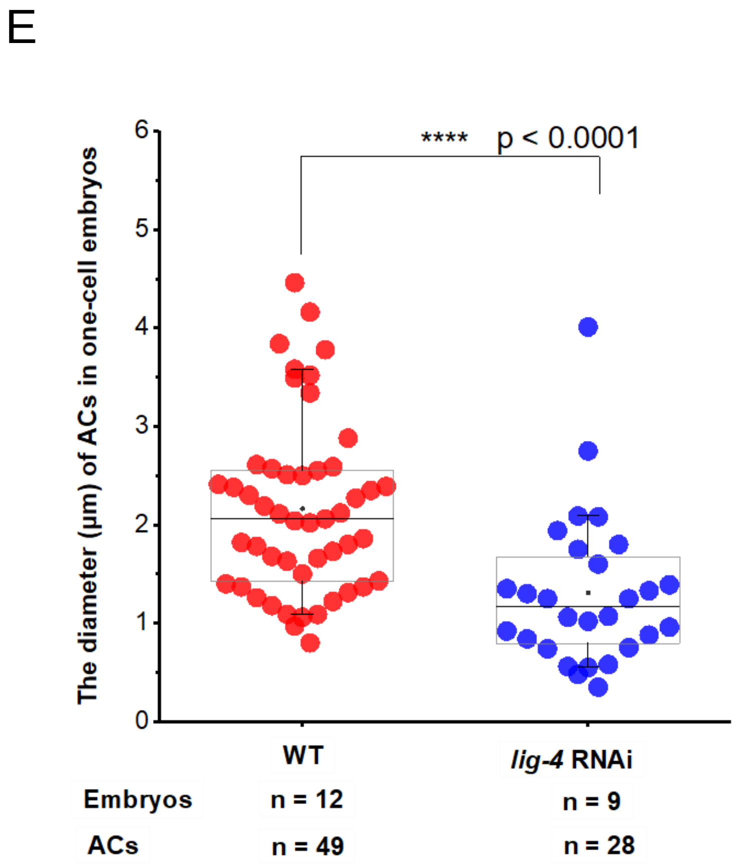
E
**** p < 0.0001
The diameter (μm) of ACs in one-cell embryos
0
1
2
3
4
5
6
WT
lig-4 RNAi
Embryos n = 12 n = 9
ACs n = 49 n = 28

Figure S6

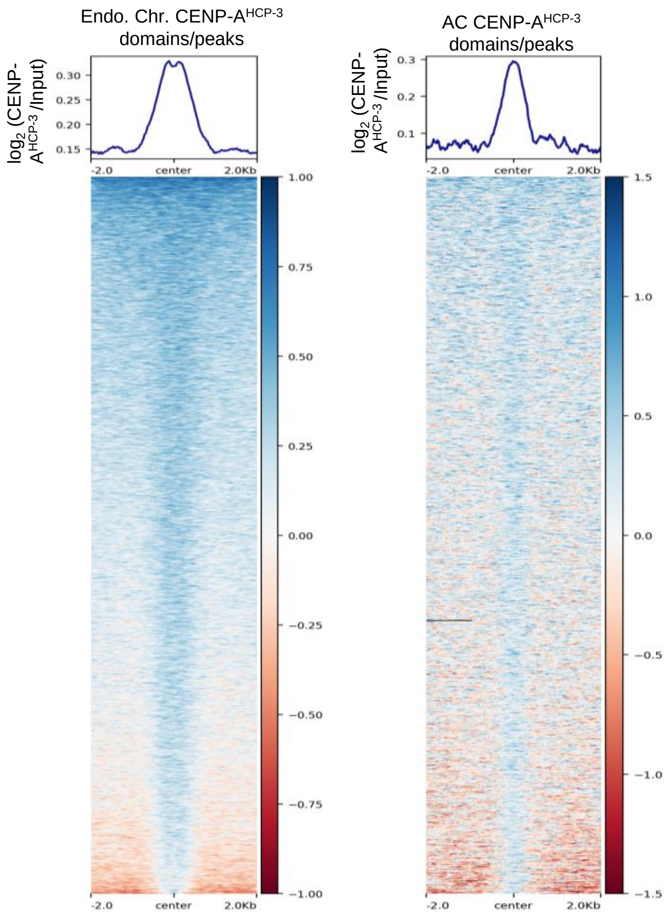

Endo. Chr. CENP-A^HCP-3 domains/peaks
AC CENP-A^HCP-3 domains/peaks
log2 (CENP-A^HCP-3 /Input)
0.30
0.25
0.20
0.15
0.3
0.2
0.1
-2.0
center
2.0Kb
1.00
0.75
0.50
0.25
0.00
−0.25
−0.50
−0.75
−1.00
1.5
1.0
0.5
0.0
−0.5
−1.0
−1.5

Figure S7

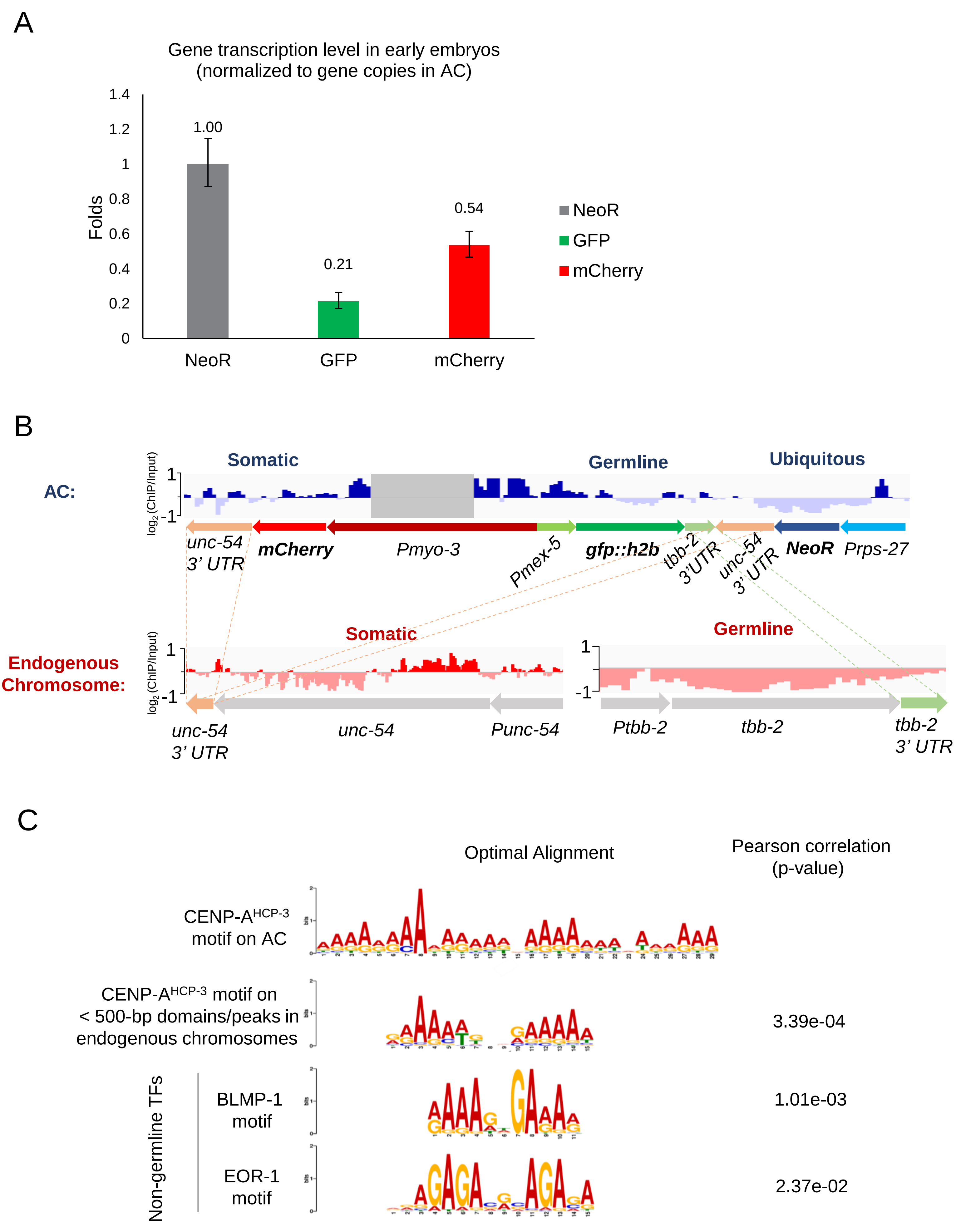
A
Gene transcription level in early embryos
(normalized to gene copies in AC)
Folds
1.4
1.2
1
0.8
0.6
0.4
0.2
0
1.00
0.21
0.54
NeoR
GFP
mCherry
B
AC:
Somatic
Germline
Ubiquitous
log2 (ChIP/Input)
unc-54 3' UTR
mCherry
Pmyo-3
Pmex-5
gfp::h2b
tbb-2 3'UTR
unc-54 3' UTR
NeoR
Prps-27
Endogenous Chromosome:
unc-54 3' UTR
unc-54
Punc-54
Ptbb-2
tbb-2
tbb-2 3' UTR
C
Optimal Alignment
Pearson correlation (p-value)
CENP-A^HCP-3 motif on AC
CENP-A^HCP-3 motif on < 500-bp domains/peaks in endogenous chromosomes
3.39e-04
Non-germline TFs
BLMP-1 motif
1.01e-03
EOR-1 motif
2.37e-02

D

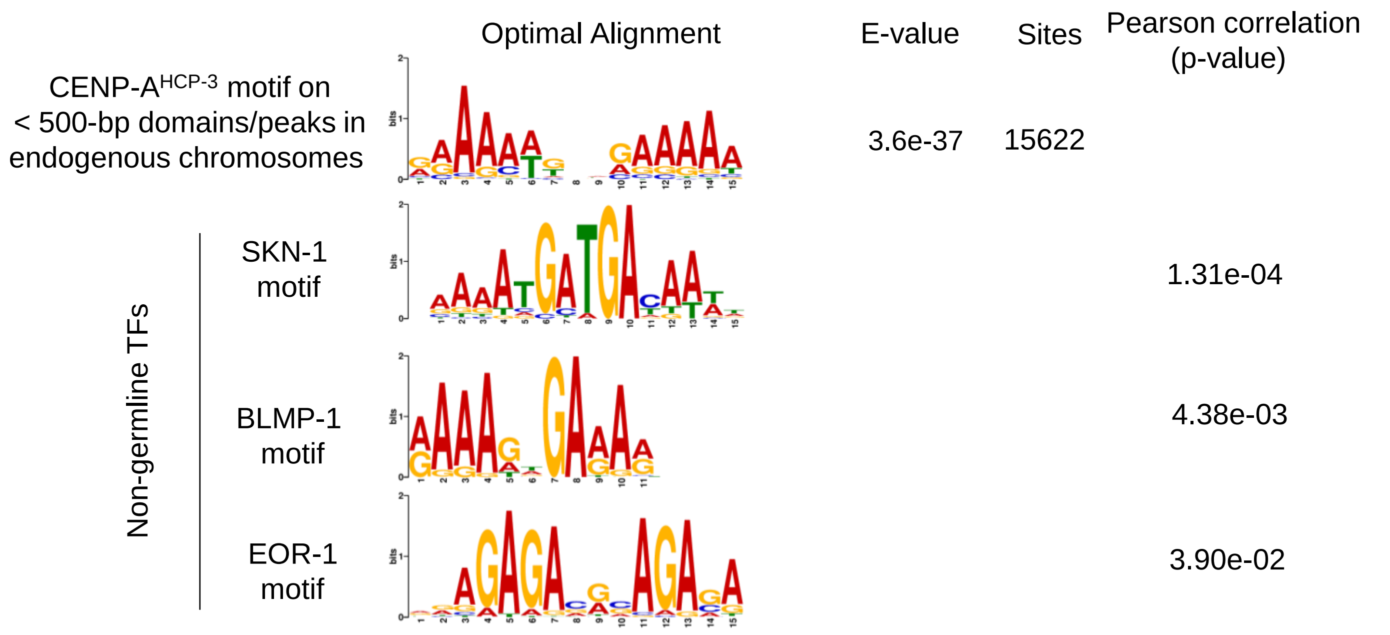
Optimal Alignment
E-value
Sites
Pearson correlation (p-value)
CENP-A^HCP-3 motif on < 500-bp domains/peaks in endogenous chromosomes
3.6e-37
15622
Non-germline TFs
SKN-1 motif
1.31e-04
BLMP-1 motif
4.38e-03
EOR-1 motif
3.90e-02

E

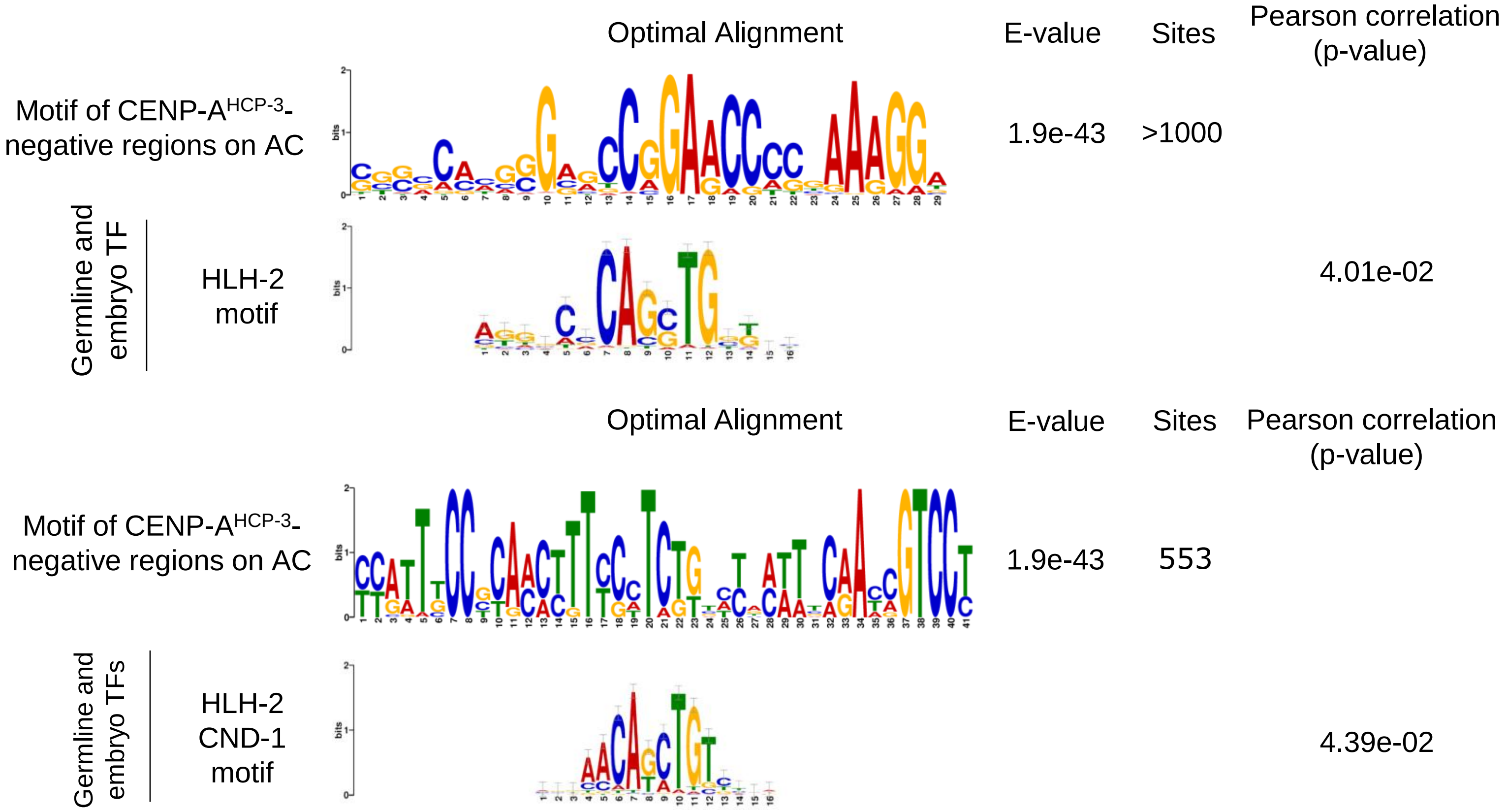
Optimal Alignment
E-value
Sites
Pearson correlation (p-value)
Motif of CENP-A^HCP-3-negative regions on AC
1.9e-43
>1000
Germline and embryo TF
HLH-2 motif
4.01e-02
Optimal Alignment
E-value
Sites
Pearson correlation (p-value)
Motif of CENP-A^HCP-3-negative regions on AC
1.9e-43
553
Germline and embryo TFs
HLH-2 CND-1 motif
4.39e-02

Figure S8

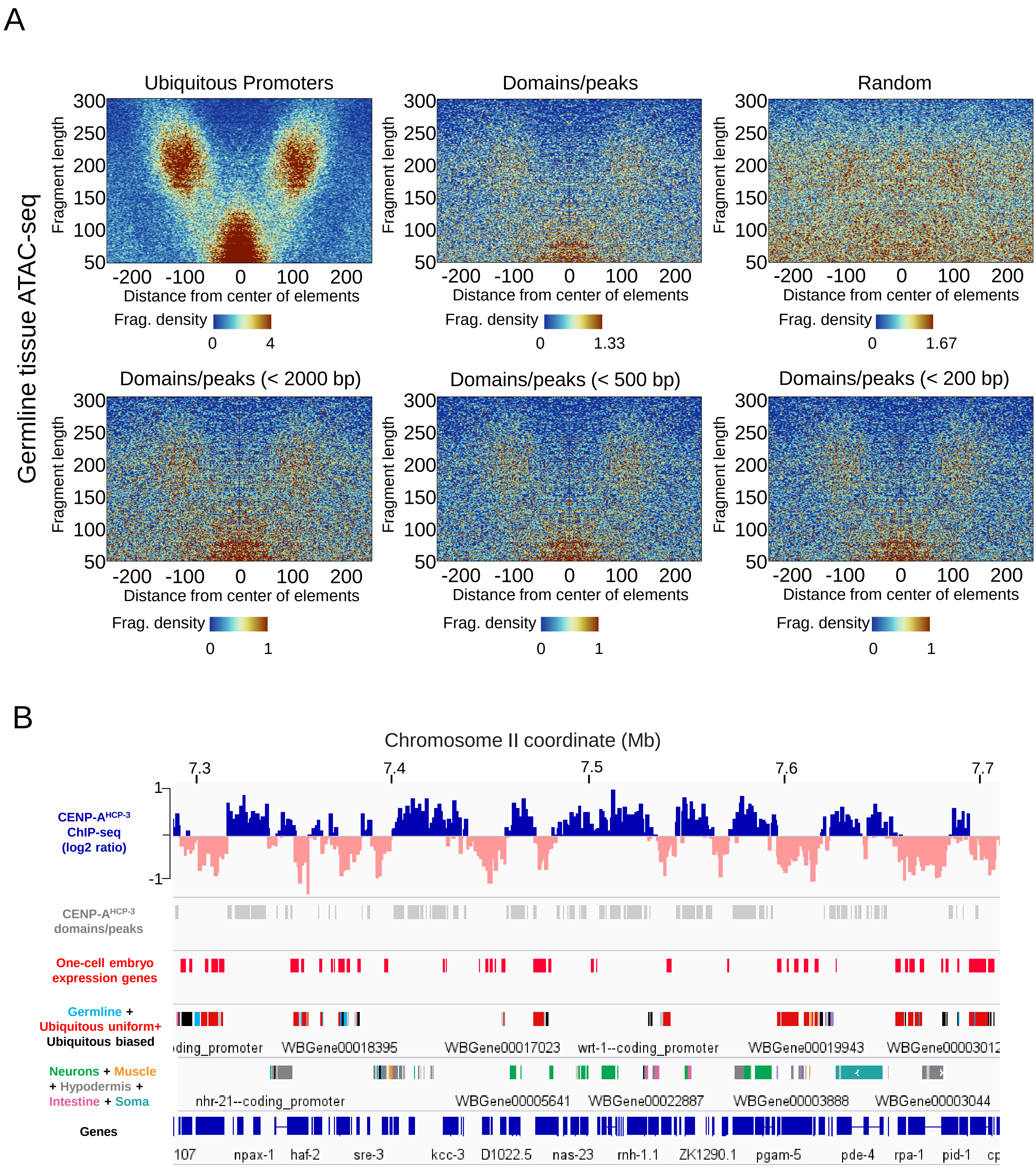

A
Germline tissue ATAC-seq
Ubiquitous Promoters
Domains/peaks
Random
Domains/peaks (< 2000 bp)
Domains/peaks (< 500 bp)
Domains/peaks (< 200 bp)
Fragment length
300
250
200
150
100
50
-200 -100 0 100 200
Distance from center of elements
Frag. density
0 4
0 1.33
0 1.67
0 1
B
Chromosome II coordinate (Mb)
7.3
7.4
7.5
7.6
7.7
CENP-AHCP-3 ChIP-seq (log2 ratio)
1
-1
CENP-AHCP-3 domains/peaks
One-cell embryo expression genes
Germline + Ubiquitous uniform+ Ubiquitous biased
Neurons + Muscle + Hypodermis + Intestine + Soma
Genes
ding_promoter
WBGene00018395
WBGene00017023
wrt-1--coding_promoter
WBGene00019943
WBGene0000301
nhr-21--coding_promoter
WBGene00005641
WBGene00022887
WBGene00003888
WBGene00003044
107
npax-1
haf-2
sre-3
kcc-3
D1022.5
nas-23
rnh-1.1
ZK1290.1
pgam-5
pde-4
rpa-1
pid-1
cp

C

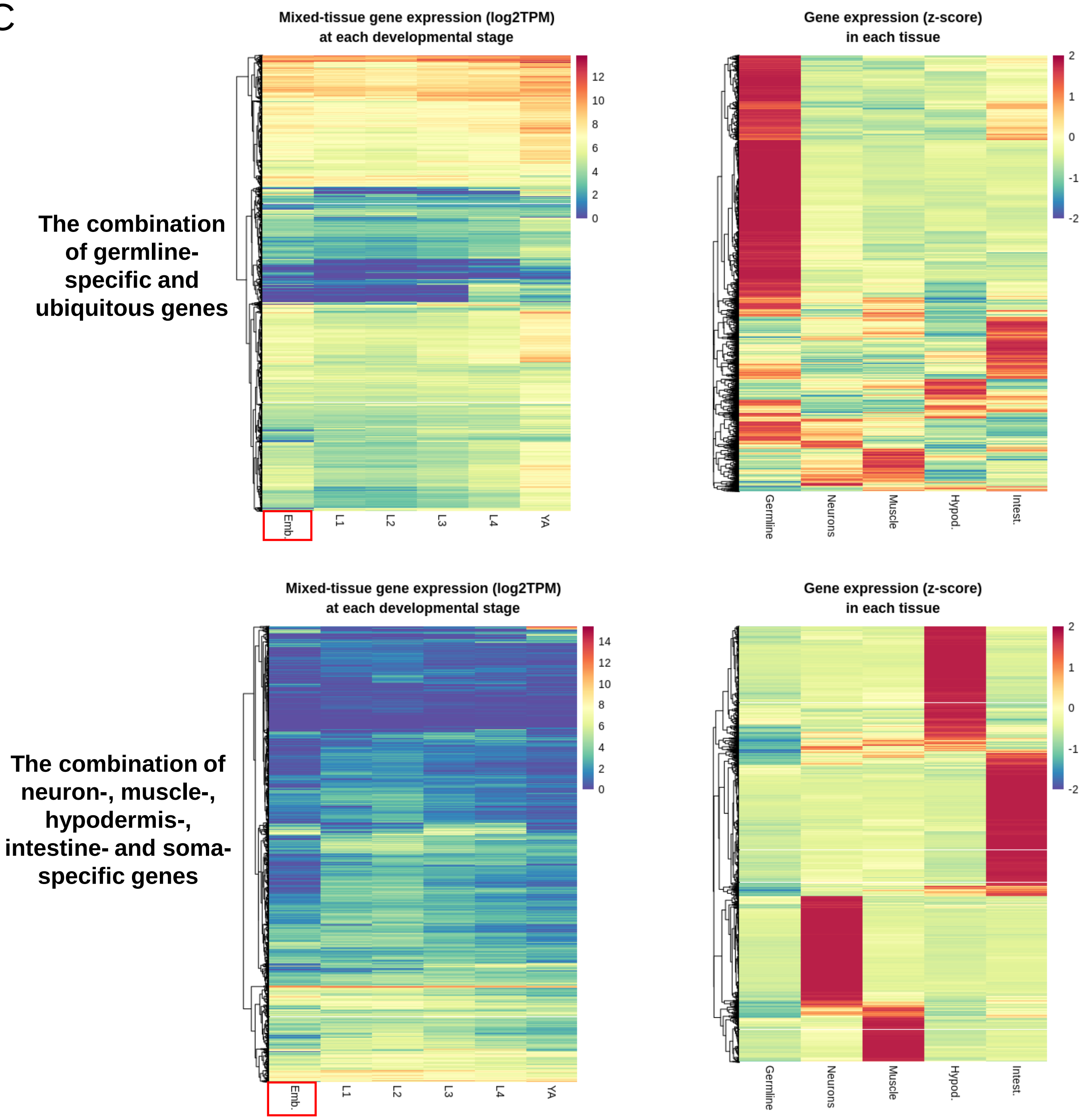

Mixed-tissue gene expression (log2TPM)
at each developmental stage
12
10
8
6
4
2
0
Emb.
L1
L2
L3
L4
YA
The combination of germline-specific and ubiquitous genes
Gene expression (z-score)
in each tissue
2
1
0
-1
-2
Germline
Neurons
Muscle
Hypod.
Intest.
Mixed-tissue gene expression (log2TPM)
at each developmental stage
14
12
10
8
6
4
2
0
Emb.
L1
L2
L3
L4
YA
The combination of neuron-, muscle-, hypodermis-, intestine- and soma-specific genes
Gene expression (z-score)
in each tissue
2
1
0
-1
-2
Germline
Neurons
Muscle
Hypod.
Intest.
